# Kalman Filter-Based RAIM for Reliable Aircraft Positioning with GPS and NavIC Constellations

**DOI:** 10.3390/s20226606

**Published:** 2020-11-18

**Authors:** Susmita Bhattacharyya, Dinesh Mute

**Affiliations:** Department of Aerospace Engineering, Indian Institute of Technology Kharagpur, Kharagpur 721302, India; dineshmute@iitkgp.ac.in

**Keywords:** RAIM, GNSS, NavIC, GPS, Kalman filter, weighted least squares

## Abstract

This paper presents a novel Kalman filter (KF)-based receiver autonomous integrity monitoring (RAIM) algorithm for reliable aircraft positioning with global navigation satellite systems (GNSS). The presented method overcomes major limitations of the authors’ previous work, and uses two GNSS, namely, Navigation with Indian Constellation (NavIC) of India and the Global Positioning System (GPS). The algorithm is developed in the range domain and compared with two existing approaches—one each for the weighted least squares navigation filter and KF. Extensive simulations were carried out for an unmanned aircraft flight path over the Indian sub-continent for validation of the new approach. Although both existing methods outperform the new one, the work is significant for the following reasons. KF is an integral part of advanced navigation systems that can address frequent loss of GNSS signals (e.g., vector tracking and multi-sensor integration). Developing KF RAIM algorithms is essential to ensuring their reliability. KF solution separation (or position domain) RAIM offers good performance at the cost of high computational load. Presented range domain KF RAIM, on the other hand, offers satisfactory performance to a certain extent, eliminating a major issue of growing position error bounds over time. It requires moderate computational resources, and hence, shows promise for real-time implementations in avionics. Simulation results also indicate that addition of NavIC alongside GPS can substantially improve RAIM performance, particularly in poor geometries.

## 1. Introduction

In recent years, global navigation satellite systems (GNSS) have evolved into important infrastructure of modern society, with day-to-day activities increasingly relying on their positioning, navigation and timing services [1,2]. GNSS is now so deeply entrenched in everyday life that disruption or malfunction of its operations can lead to undesirable consequences. Among many other diverse applications, aircraft navigation in particular has immensely benefited from GNSS during different flight phases (e.g., cruising, terminal, and approach to landing) [3,4,5,6]. GNSS is also identified as a key sensor on-board unmanned aerial vehicles (UAV) [7,8,9]. In spite of ever expanding use, major concerns in aviation applications remain to be reliability and continuity of GNSS services. The reason is that a system anomaly/loss of signal in aviation can jeopardize safety, or cause legal or economic liabilities.

**Figure 1:**
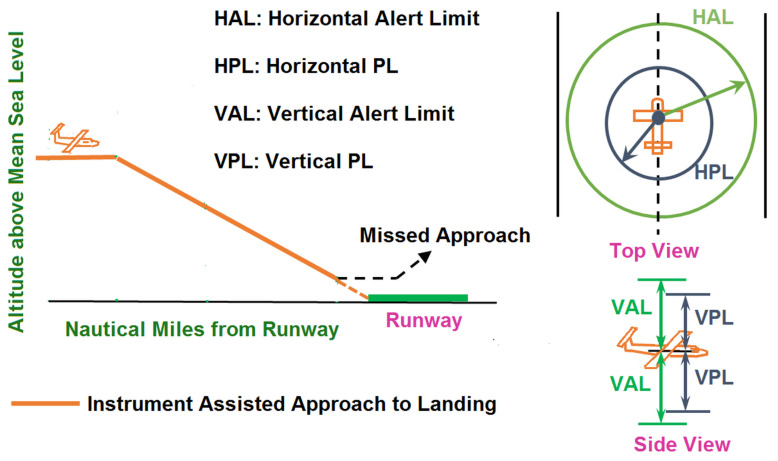
Illustration of PLs. To ensure reliability, magnitude of error in the estimated position (both horizontal and vertical) must be within the corresponding PL with a certain probability. Calculated PLs must be below the alert limits when integrity monitoring is available.

In order to guarantee reliability, a GNSS signal integrity monitor is integrated with avionics. Integrity is considered an essential performance metric in aviation. It is a measure of trustworthiness of a system [10]. An integrity monitor is designed to issue alarms to pilots within a prescribed time, when reliable GNSS performance cannot be ensured. To this end, it carries out continuous fault detection tests and raises an alarm in case a fault is detected. If no fault is identified, the monitor outputs a bound on position error named protection level (PL). The PL protects error in estimated position with a large probability. As an example, Figure 1 illustrates PLs for an approach to landing application.

Design of integrity monitors (or monitoring algorithms) relies on what navigation filter is used for GNSS signal processing. Two standard candidates are weighted least squares (WLS) and Kalman filter (KF) (or in particular, extended KF (EKF)). WLS is easy to implement, but not ideal for advanced navigation systems [11]. On the other hand, EKF can be used in traditional as well as advanced methods. It is an important part of advanced systems (e.g., vector tracking [12] and multi-sensor integration [13]). These systems are more accurate and robust. They can address the vulnerability of weak GNSS signals, which are prone to loss of lock in the presence of moderate to high dynamics [14,15,16,17]. Thus, advanced navigation systems can help improve continuity of GNSS services.

While integrity monitoring with WLS is extensively studied and well-developed [18,19,20,21,22,23,24,25], relatively few studies are available on its KF counterpart [26,27,28,29,30,31,32,33,34,35,36,37,38]. To this end, a novel KF-based integrity algorithm is designed in this paper for reliable absolute positioning with GNSS pseudorange (carrier smoothed) and pseudorange rate measurements. The presented method, being computationally efficient, shows promise for real-time implementations in avionics. It also has potential for extensions to advanced methods (e.g., vector tracking [30]), thereby contributing to enhancing both reliability and continuity of GNSS operations. The algorithm’s performance is compared with two existing approaches—one each for WLS and KF—for a UAV navigation scenario.

Over the last two decades, extensive research has been carried out on integrity monitoring with the Global Positioning System (GPS) in civil aviation [21,25]. In order to meet stringent requirements, dual frequency multi-constellation GNSS signals were deemed necessary [23]. Consequently, there are studies on combined integrity performance of GPS and Galileo/Beidou in the context of aviation [24,39]. However, to the best of the authors’ knowledge, no such research is available in the open literature in relation to the Indian GNSS, Navigation with Indian Constellation (NavIC). This navigation satellite constellation provides coverage to the Indian sub-continent and areas extending about 1500 km around it [40,41]. NavIC is intended to support aviation users among others over its coverage area. With regard to this, the current work aims to analyse KF integrity performance with GPS and NavIC. Design of the algorithm is also discussed with a focus on the dual constellations. However, the methodology can apply to other GNSS systems with appropriate changes.

The organization of the remaining paper is as follows: First, prior work on integrity algorithms is discussed. Contributions of the current work are also noted in this context. Following this, a brief overview of existing KF-based integrity algorithms is provided. Next, the integrity monitoring algorithm proposed in this paper is described. Subsequently, the performance of the KF integrity algorithm is compared with two other approaches using simulated GPS and NavIC measurements for a UAV trajectory. Finally, the paper ends with conclusions and future extensions of the current work.

## 2. Prior Work and Contributions

Aircraft-based augmentation system (ABAS) is one of the different methods implemented for GNSS integrity monitoring for aviation users. It has a built-in integrity monitor in avionics. An integral part of ABAS is a GNSS receiver-based algorithm called receiver autonomous integrity monitoring or RAIM [3]. A detailed literature survey of RAIM algorithms for civil aviation is presented in [10]. As mentioned before, RAIM depends on what navigation filter (WLS or KF) is used in the GNSS signal processing of the receiver. An extensive body of knowledge is available in the literature on WLS RAIM [20,21]. Position domain implementation of WLS RAIM is called solution separation method. Advanced RAIM (ARAIM) extends this method by including dual frequency, multi-constellation GNSS signals, an extensive threat model and an integrity support message [42]. ARAIM is envisioned to support horizontal and vertical guidance of aircraft. On the other hand, range domain implementation of RAIM is called range-based RAIM in this paper. Its formulation for WLS dates back to the late 1980s and early 1990s [18,19]. This method has been extended to handle multiple faults in [20]. RAIM for a batch least squares algorithm is developed in [22].

Solution separation RAIM for KF was first presented in [26] for aviation applications. It performs integrity monitoring in the position domain by eliminating a visible satellite from one of multiple KFs running in parallel. References [27,28] extend ARAIM to KF for high accuracy and precision applications, and propose methods to reduce the computational load for running parallel filters. In [29], a range-based KF RAIM algorithm is described. It develops a recursive fault detection test with KF residuals, and calculates the worst-case fault vector using a batch least squares approach. Range-based KF RAIM has an advantage over position domain method in that it does not need parallel filters. It has less architectural complexity, but it needs to take into account contributions from past epoch measurements to the current PL. This should be done in a computationally efficient way without increasing the PL as time progresses [31]. Reference [32], on the other hand, proposes an isotropy-based RAIM algorithm with KF.

Integrity monitoring for integrated GNSS-inertial navigation system (INS) was recently studied in [34,35,36,37,38] in both range and position domains for KF. In [34] an integrity monitor is presented in the range domain to deal with spoofing attacks during an aircraft approach to a runway. The work is later extended with a computationally efficient calculation of the worst-case fault vector [35]. In a subsequent reference [36], solution separation approach is preferred over the proposed range-based monitor in case of good satellite visibility for an autonomous land vehicle application. Solution separation RAIM for tightly coupled GNSS-INS is described in [37] for precise point positioning. A fault detection and exclusion scheme for tightly coupled GNSS-INS system is designed in [38]; it can detect multiple GNSS, and simultaneous GNSS and INS faults.

Restricting the increase of PLs over time in range-based KF RAIM is still a major challenge, with little work done in this regard. In [33], an implementation of range domain KF RAIM is provided for GNSS receivers. Its PL does not grow with time due to contributions from previous measurements to the present epoch. However, it assumes a single satellite failure, and does not model time correlated errors of measurements. A range-based KF RAIM algorithm is detailed in this paper, building upon [33]. It models time correlated errors, and handles multi-satellite failures. The algorithm also retains computational efficiency. A different implementation of KF called Schmidt KF (SKF) [43] is required for design. It is essential to the formation of fault detection tests. More precisely, it helps in overcoming the issue of growing PLs by enabling more than one test in the presence of time correlated errors. Each test considers measurement innovations of a finite number of epochs, as before, but requires a completely different formulation. Hence, the PL computation is also significantly modified. It should be noted that constellation-wide faults due to a common cause are not considered in the current work. Measurements of a given epoch are also assumed to be uncorrelated with each other.

Extensive simulations over the primary coverage area of NavIC on the Indian sub-continent were performed for a 20-min UAV trajectory. With GPS and NavIC measurements, it is shown that the existing methods used as reference outperform the new one. However, the work is significant for the following reasons. KF is an integral part of advanced navigation systems, as noted earlier. Therefore, developing KF RAIM algorithms is essential to ensuring their reliability. The improvement of KF solution separation RAIM is obtained at the expense of high computational load. The presented range-based KF RAIM, on the other hand, offers satisfactory performance to a certain extent with moderate computational resources. It has potential for extensions to advanced methods such as vector receivers, where running parallel vector tracking loops for solution separation RAIM would be too complex and prohibitively expensive. Further, simulation results indicate that the addition of NavIC alongside GPS can substantially improve performance, particularly in poor geometries.

## 3. Overview of Existing KF RAIM Algorithms

The developed KF RAIM is compared with two existing integrity algorithms. The first one is range-based RAIM with WLS [20]. Although snapshot in nature, it is chosen for performance comparison because it works in the range domain, like the presented method. The second one, KF RAIM in the position domain, implements the solution separation approach with the EKF. Since the WLS RAIM algorithm is described in detail in [20], it is not repeated here. First, a brief overview of existing solution separation KF RAIM is provided. Following this, background of the existing range-based KF RAIM, which is extended further later in this paper, is presented for ease of understanding.

### 3.1. Solution Separation KF RAIM

First, error models and key integrity parameters are described. Although these are discussed in the context of solution separation method, these are used by all RAIM algorithms of this paper. The following error models are considered. Ionospheric error is assumed to be removed using dual frequency measurements. Broadcast satellite clock and ephemeris error, residual tropospheric delay (i.e., remaining error after removing the modeled component), noise and multipath in pseudorange measurements are modeled as a first order Gauss Markov (GM) process with 100 s time constant. Their standard deviations are taken from [44,45] for carrier smoothed code pseudorange measurements of GPS in L${}_{1}$ and L${}_{5}$ bands for airborne users. More realistic error models with different time constants for individual components will be considered in future work. Due to lack of availability of similar error models for NavIC, the same equations as those for GPS are assumed with frequency bands appropriately changed. Only white noise is modeled in the pseudorange rate measurements. Noise standard deviation corresponds to a C/N${}_{0}$ of 45 dB-Hz.

The prior probability of narrow fault $P_{F}$ for both GPS and NavIC constellations is assumed to be $10^{-4}$/satellite/h. Over the years, $P_{F}$ is found to be less than this value for GPS [42]. For NavIC being a new constellation, many years of data are yet to be analyzed before arriving at a suitable probability. Constellation-wide faults are not considered in this work. The allocated probability of hazardously misleading information (HMI) (i.e., the probability that position error exceeds the PL but no alert is issued), $P_{HMI}^{alloc}$, is considered $10^{-7}$/h [46]. $P_{HMI}^{alloc}$ for the vertical component, $P_{HMI,\;V}^{alloc}$ is $9\times 10^{-8}$/h (i.e., 90% of $P_{HMI}^{alloc}$). $P_{HMI}^{alloc}$ for the horizontal component, $P_{HMI,\;H}^{alloc}$ is $1\times 10^{-8}$/h. For the assumed $P_{HMI}^{alloc}$ (or allocated integrity budget) and $P_{F}$, monitoring up to two simultaneous independent satellite failures is sufficient, as discussed in [24]. This is because three or more simultaneous failures will have a probability less than the total integrity budget. The probability of three or more unmonitored faults $P_{um}$ is therefore subtracted from the integrity budget. The continuity budget allocated to disruptions due to false alert $P_{FA}$ is $3.33\times 10^{-7}$/sample [47]. $P_{FA}$ and $P_{HMI}^{alloc}$ values chosen correspond to the en route phase of civil aviation.

Let $P_{HMI,\;h}^{nf}$ be the probability of HMI for no-fault (NF) condition, where *h* = 1, 2, 3 for north, east and down components of position, respectively. $P_{HMI,\;h}^{sf}$ is the probability for single-fault (SF) modes. $P_{HMI,\;h}^{df}$ is the probability for dual-fault (DF) modes. For each horizontal component of position, their allocated values are

(1) $$\begin{array}{cc} P_{HMI,\;h}^{alloc,\;nf} & =1.8\times 10^{-11}/h,\;P_{HMI,\;h}^{alloc,\;sf}=1.8\times 10^{-11}/h,\\ P_{HMI,\;h}^{alloc,\;df} & =4.849\times 10^{-9}/h \end{array}$$

where *h* = 1 and 2 denote north and east components, respectively. NavIC constellation currently has seven operational satellites in orbit. Assuming a maximum of twelve GPS satellites, the sum of the preceding probabilities is slightly less than half of $(P_{HMI,\;H}^{alloc}-0.1P_{um})$.

For the vertical component of position, the allocated probabilities are

(2) $$\begin{array}{cc} P_{HMI,\;3}^{alloc,\;nf} & =3.6\times 10^{-10}/h,\;P_{HMI,\;3}^{alloc,\;sf}=3.6\times 10^{-10}/h,\\ P_{HMI,\;3}^{alloc,\;df} & =8.825\times 10^{-8}/h \end{array}$$

The sum of the above three probabilities is a little less than $(P_{HMI,\;V}^{alloc}-0.9P_{um})$. The probabilities are not optimally chosen to minimize PLs due to higher computational load involved in optimization. As an alternative, a larger value (about 99% of the total probability) is allocated to dual-satellite fault mode instead of assigning one third of the sum to each of NF, SF and DF. This is to reduce PLs as the DF mode is found to result in higher PLs.

Position domain KF RAIM implemented in this paper adapts solution separation approach to EKF. It runs parallel EKFs—one for each fault mode plus an all-in-view filter for NF condition. Each EKF estimates errors in three coordinates of aircraft position and velocity in the Earth centered Earth Fixed (ECEF) frame, and receiver clock biases and drifts for GPS and NavIC from their respective a priori values. In addition, each of them has additional states (one for each pseudorange measurement) to model time correlated errors as first order GM processes. Key equations of KF solution separation RAIM are provided in Appendix A. Satellite position, velocity and modeled tropospheric delays are calculated only in the all-in-view filter and shared among all subset filters—one for each fault mode—to reduce the computational load. State error covariances are updated using a numerically stable forward recursive modified rank-one update algorithm [43]. Next, an overview of existing range-based KF RAIM is provided to lay foundation for the modified algorithm described later in this paper.

### 3.2. Existing Range-Based KF RAIM 

In the fault detection method of existing range-based RAIM, three test statistics are formed, each with KF measurement innovations of a number of epochs [33]. The reason for three tests is described in detail in the reference. The first statistic, $\alpha_{1}$, is calculated with measurement innovations of *M* epochs including the current epoch $t_{k}$. The second statistic, $\alpha_{2}$, has (N−M) epochs from k−N+1 to k−M. The third test statistic, $\alpha_{3}$, is formed with innovations of all epochs before *N* epochs, whose contributions are included in $\alpha_{3}$ before these terms are discarded (or removed from memory). At a time, only *N* epoch terms are retained in memory, but test statistics are formulated with innovations of all epochs. *N* and *M* are adaptively determined. Mathematically, the test statistics at $t_{k}$, ($\alpha_{j,\;k}$; *j* = 1, …, 3), are given by

(3) $$\begin{array}{cc} \alpha_{j,\;k}^{2} & =\sum_{\ell =p_{j}}^{m_{j}}\left(\Delta {\underline{\rho }}_{\ell }^{T}W_{\ell }^{-1}\left(I-G_{\ell }\right)\Delta {\underline{\rho }}_{\ell }+\Delta {\underline{\dot{\rho }}}_{\ell }^{T}{W_{rr,\;\ell }}^{-1}\left(I-G_{\ell }^{rr}\right)\Delta {\underline{\dot{\rho }}}_{\ell }\right) \end{array}$$

where $\Delta {\underline{\rho }}_{\ell }$ and $\Delta {\underline{\dot{\rho }}}_{\ell }$ denote EKF pseudorange and pseudorange rate innovation vectors, respectively, at epoch *ℓ*. $m_{j}$ and $p_{j}$ hold different values for different *j*, as mentioned before. I is the identity matrix of size n×n; and *n* is the number of visible satellites at an epoch. *n* can change with time. $G_{\ell }$ and $G_{\ell }^{rr}$ are computed by WLS estimation. They are

(4) $$\begin{array}{cc} G_{\ell } & =H_{\ell }{\left(H_{\ell }^{T}W_{\ell }^{-1}H_{\ell }\right)}^{-1}H_{\ell }^{T}W_{\ell }^{-1} \end{array}$$

(5) $$\begin{array}{cc} G_{\ell }^{rr} & =H_{\ell }{\left(H_{\ell }^{T}{W_{rr,\;\ell }}^{-1}H_{\ell }\right)}^{-1}H_{\ell }^{T}{W_{rr,\;\ell }}^{-1} \end{array}$$

where $W_{\ell }$ is the pseudorange measurement error covariance matrix at $t_{\ell }$th epoch. $H_{\ell }$ is linearized pseudorange (or pseudorange rate) measurement model matrix. $W_{rr}$ is the pseudorange rate error covariance matrix. The error model accounts for unmodeled propagation delays by inflating noise covariance, and neglects error in broadcast satellite clock terms and ephemeris, and multipath. Measurement error between time epochs is thus assumed uncorrelated. Fault detection method considers WLS estimation whereas PL calculation is based on EKF position error.

A WLS estimation-based fault detection test with EKF innovations allows simplified calculations of mean position error bounds, as discussed in the reference. With WLS estimation-based fault detection, each term of the test statistic under summation in Equation (3) and in the denominator of failure mode slope (FMS) would contribute fault of only the corresponding epoch, not any other epochs. This simplifies FMS calculation, and allows the formation of a separate FMS for mean position error bound pertaining to a test statistic. As a result, more than one test can be formulated. The noise statistics of αs can also be easily computed. It was proved in [31] that each term in Equation (3) generated with KF innovations has the same noise distribution as that of the WLS navigation filter. The threshold $T_{th,\;j}$ for an $\alpha_{j}$ is therefore computed from the Chi square distribution with probability $P_{\mathrm{FA}}/3$ and appropriate degrees of freedom (DOF). A fault is declared if at least one of the test statistics crosses its threshold.

The bounds on horizontal and vertical mean position error under a fault, HPE${}_{i}^{U}$ and VPE${}_{i}^{U}$, respectively, for a satellite *i* are derived as

(6) $$\begin{array}{cc} {\mathrm{HPE}}_{i}^{U} & =\sum_{j=1}^{3}{\mathrm{HPE}}_{i,\;j}^{U}+\sqrt{{\left({\underline{\mu }}_{n}^{\mathrm{error},\;U}\left(1\right)\right)}^{2}+{\left({\underline{\mu }}_{n}^{\mathrm{error},\;U}\left(2\right)\right)}^{2}} \end{array}$$

(7) $$\begin{array}{cc} {\mathrm{VPE}}_{i}^{U} & =\sum_{j=1}^{3}{\mathrm{VPE}}_{i,\;j}^{U}+{\underline{\mu }}_{n}^{\mathrm{error},\;U}\left(3\right) \end{array}$$

where ${\mathrm{HPE}}_{i,\;j}^{U}$ and ${\mathrm{VPE}}_{i,\;j}^{U}$ are bounds corresponding to $\alpha_{j}$ (i.e., they consider the same epoch terms as those of $\alpha_{j}$), with *j* = 1, 2, 3. ${\underline{\mu }}_{n}^{\mathrm{error},\;U}\left(1:3\right)$ is a three-element vector of north, east and down mean position error bounds resulting from second order terms of nonlinear GNSS measurement equations.

It is important to note that the DOF of $\alpha_{3}$, and ${\mathrm{HPE}}_{i,\;3}^{U}$ and VPE${}_{i,\;3}^{U}$ grow with time. However, as HPE${}_{i,\;3}^{U}$ and VPE${}_{i,\;3}^{U}$ have very small contributions, VPE${}_{i}^{U}$ and HPE${}_{i}^{U}$ do not have the same growing trend over time. It is justified through simulation results that with three test statistics and mean position error bounds as calculated in the preceding equations, PL does not grow over time, unlike the PL with a single test statistic. Faults are also detected in a shorter time than that with a single test formed using all innovation terms from the beginning up to the current epoch. The reason is that the growing DOF of a single test statistic increases the fault detection threshold fast, resulting in late detection of faults. PLs are computed using the above-mentioned mean position error bounds under the assumption of a single satellite fault.

As noted before, SKF is used as navigation processor for range domain KF RAIM algorithm developed in this paper. Its formulation is briefly provided next.

## 4. Schmidt KF

In this section, the SKF formulation of KF is briefly outlined. Interest readers can refer to [43,48] for more details. The reason for using SKF will be explained in the next section while discussing the fault detection test of range-based KF RAIM. Although sub-optimal in nature, SKF accounts for the effects of time correlated errors without estimating additional states. It also has fast execution time.

It is desired to determine aircraft absolute position and velocity, and receiver clock biases and drifts for GPS and NavIC. Their true values and a priori estimate are denoted as $\underline{\varkappa }$ and ${\tilde{\underline{\varkappa }}}^{-}$, respectively. The difference between $\underline{\varkappa }$ and ${\tilde{\underline{\varkappa }}}^{-}$, $\Delta {\underline{\varkappa }}_{k}$ is estimated. Thus, the SKF state vector at $t_{k}$ is

(8) $$\Delta {\underline{\varkappa }}_{k}={\left[\Delta x\;\Delta \dot{x}\;\Delta y\;\Delta \dot{y}\;\Delta z\;\Delta \dot{z}\;\Delta b_{\mathrm{clk}}^{G}\;\Delta {\dot{b}}_{\mathrm{clk}}^{G}\;\Delta b_{\mathrm{clk}}^{N}\;\Delta {\dot{b}}_{\mathrm{clk}}^{N}\right]}_{k}^{T}$$

where x,yandz are three position components of aircraft expressed in ECEF coordinate frame. $\dot{x}$, $\dot{y}$ and $\dot{z}$ are velocity components in ECEF. $b_{\mathrm{clk}}^{G}$ and $b_{\mathrm{clk}}^{N}$ are the receiver clock biases for GPS and NavIC, respectively, and ${\dot{b}}_{\mathrm{clk}}^{G}$ and ${\dot{b}}_{\mathrm{clk}}^{N}$ are the receiver clock drifts for the two constellations.

Broadcast ephemeris and clock error, residual tropospheric delay along the line of sight, multipath and noise in carrier smoothed code pseudorange measurements of a satellite are modeled as a first order GM process with time constant 100 s. Let $\sigma_{\mathrm{ura}}^{i}$, $\sigma_{\mathrm{tr}}^{i}$, $\sigma_{\mathrm{mp}}^{i}$ and $\sigma_{n}^{i}$ be the standard deviations of broadcast ephemeris and clock error, residual tropospheric delay, multipath and noise, respectively, for *i*th satellite. Their elevation angle dependent models for airborne users are taken from [44,45] for GPS $L_{1}$ and $L_{5}$ band measurements and provided next.

(9) $$\begin{array}{cc} \sigma_{\mathrm{tr}}^{i} & =1.001/\sqrt{0.002001+{\left(\sin\left(\theta_{i}\right)\right)}^{2}}\times 0.12 \end{array}$$

(10) $$\begin{array}{cc} \sigma_{\mathrm{mp}}^{i} & =\left(0.13+0.53\exp^{-(\theta_{i}\times 180/\pi /10)}\right)\sqrt{\frac{\left(1.57542^{4}+1.17645^{4}\right)}{{\left(1.57542^{2}-1.17645^{2}\right)}^{2}}} \end{array}$$

(11) $$\begin{array}{cc} \sigma_{n}^{i} & =\left(0.15+0.43\exp^{-(\theta_{i}\times 180/\pi /6.9)}\right)\sqrt{\frac{\left(1.57542^{4}+1.17645^{4}\right)}{{\left(1.57542^{2}-1.17645^{2}\right)}^{2}}} \end{array}$$

where $\theta_{i}$ is the elevation angle of *i*th GPS satellite in radians. $\sigma_{\mathrm{ura}}$ is assumed to be 0.75. Thus, the resultant error is a GM process with 100 s time constant, and has a standard deviation of $\sqrt{{\left(\sigma_{\mathrm{ura}}^{i}\right)}^{2}+{\left(\sigma_{\mathrm{tr}}^{i}\right)}^{2}+{\left(\sigma_{\mathrm{mp}}^{i}\right)}^{2}+{\left(\sigma_{n}^{i}\right)}^{2}}$ for *i*th GPS satellite. The same error models are used for NavIC, but the frequencies are changed to $L_{5}$ and *S* bands.

Pseudorange rate measurement error is modeled as white Gaussian noise with standard deviation corresponding to a C/N${}_{0}$ of 45 dB-Hz. Thus, there are *n* GM processes, where *n* is the number of visible satellites at an epoch. In the SKF algorithm, states of GM processes are not estimated, but their effects are considered. If the vector ${\underline{p}}_{gm,\;k-1}$ has the states of *n* GM processes at $t_{k-1}$, then $\Delta {\underline{\varkappa }}_{k-1}$ and ${\underline{p}}_{gm,\;k-1}$ can be combined as ${\underline{X}}_{k-1}$ = ${\left[\Delta {\underline{\varkappa }}_{k-1}^{T}\;{\underline{p}}_{gm,\;k-1}^{T}\right]}^{T}$. The process and linearized measurement models of ${\underline{X}}_{k}$ and measurement update equations of SKF are described in Appendix B. Next, the range-based KF RAIM algorithm is presented.

## 5. Range-Based KF RAIM Algorithm

First, the fault detection method in the presence of time correlated errors is developed. Then, mean position error bounds and PLs are computed considering up to two simultaneous independent satellite failures.

### 5.1. Fault Detection Method

The test statistic described by Equation (3) earlier is modified to account for time correlated errors in pseudorange measurements, as explained next. Since no time correlated error is modeled in the pseudorange rate measurements, part of the test statistics with pseudorange rate innovations remains the same as before. It can be shown that

(12) $$\Delta {\underline{\rho }}_{\ell }^{T}W_{\ell }^{-1}\left(I-G_{\ell }\right)\Delta {\underline{\rho }}_{\ell }={\Delta {\underline{\rho }}^{*}}_{\ell }^{T}\left(I-G_{\ell }^{*}\right){\Delta {\underline{\rho }}^{*}}_{\ell }$$

where ${\Delta {\underline{\rho }}^{*}}_{\ell }^{T}$ = $W_{\ell }^{-1/2}\Delta {\underline{\rho }}_{\ell }^{T}$ and $G_{\ell }^{*}$ = $W_{\ell }^{-1/2}H_{\ell }{\left(H_{\ell }^{T}W_{\ell }^{-1}H_{\ell }\right)}^{-1}\times H_{\ell }^{T}W_{\ell }^{-1/2}$ where ‘×’ is matrix multiplication. Using the idempotent property of $\left(I-G_{\ell }^{*}\right)$ [18]

(13) $${\Delta {\underline{\rho }}^{*}}_{\ell }^{T}\left(I-G_{\ell }^{*}\right){\Delta {\underline{\rho }}^{*}}_{\ell }={\Delta {\underline{\rho }}^{*}}_{\ell }^{T}\left(I-G_{\ell }^{*}\right)\left(I-G_{\ell }^{*}\right){\Delta {\underline{\rho }}^{*}}_{\ell }$$

With singular value decomposition, $\left(I-G_{\ell }^{*}\right)$ can be written as

(14) $$\left(I-G_{\ell }^{*}\right)=S_{\ell }LS_{1\ell }$$

L is a diagonal matrix. Its diagonal elements are ones and zeros. The number of zeros is equal to the dimension of the null space of $\left(I-G_{\ell }^{*}\right)$. For a single constellation it is four whereas for dual constellations with a separate receiver clock bias state for each, it is five. Let the null space dimension be denoted as $n_{\mathrm{null}}$. It can be shown that $\left(I-G_{\ell }^{*}\right)$ is also equal to

(15) $$\left(I-G_{\ell }^{*}\right)=S_{\ell }\left(1:n,1:n-n_{\mathrm{null}}\right){\left(S_{\ell }\left(1:n,1:n-n_{\mathrm{null}}\right)\right)}^{T}$$

where $J(1:n_{1},1:n_{2})$ represents first $n_{1}$ rows and $n_{2}$ columns of a matrix J. Using Equations (12) and (13), and replacing each $\left(I-G_{\ell }^{*}\right)$ in Equation (13) with Equation (15) and $S_{\ell }\left(1:n,1:n-n_{\mathrm{null}}\right)$ with $S_{\ell }^{\prime }$, part of the test statistic formed with pseudorange innovations, $\sum_{\ell =p_{j}}^{m_{j}}\Delta {\underline{\rho }}_{\ell }^{T}W_{\ell }^{-1}\left(I-G_{\ell }\right)\Delta {\underline{\rho }}_{\ell }^{T}$ (denoted as ${\left(\alpha_{j}^{\prime }\right)}^{2}$ later) in Equation (3) is changed as

(16) $$\begin{array}{c} {\left(\alpha_{j}^{\prime }\right)}^{2}={\Delta {\underline{\tilde{\rho }}}^{*}}^{T}{\tilde{S}}^{\prime }{{\tilde{S}}^{\prime }}^{T}\Theta {\tilde{S}}^{\prime }{{\tilde{S}}^{\prime }}^{T}\Delta {\underline{\tilde{\rho }}}^{*} \end{array}$$

where $\Delta {\underline{\tilde{\rho }}}^{*}$ stacks all $\Delta {\underline{\rho }}_{\ell }^{*}$ vectors with *ℓ* varying from the lower limit $p_{j}$ to the upper limit $m_{j}$ of Equation (3). ${\tilde{S}}^{\prime }$ is a block matrix having non-zero sub-matrices (or blocks) along the diagonal, with the *ℓ*th block given by $S_{\ell }^{\prime }$. Θ is a square matrix with number of rows/columns equal to $\sum_{\ell =p_{j}}^{m_{j}}n$. It is introduced in the middle and defined later. When it is the identity matrix, Equation (16) reduces to Equation (3). For ease of representation, the time epoch *k* is dropped from the subscript. The mathematical model of $\Delta {\underline{\tilde{\rho }}}^{*}$ is given by

(17) $$\Delta {\underline{\tilde{\rho }}}^{*}=H^{*}\Delta {\underline{\varkappa }}_{p}+{\underline{\eta }}_{1}$$

$H^{*}$ is a block matrix with non-zero blocks along the diagonal. Its *ℓ*th block is $W_{\ell }^{-1/2}H_{\ell }$. $H_{\ell }$ is defined after Equation (5). Only difference is that $H_{\ell }$ has an additional column for receiver clock bias corresponding to the second constellation. $\Delta {\underline{\varkappa }}_{p}$ stacks all ${\left[\Delta x\;\Delta y\;\Delta z\;\Delta b_{\mathrm{clk}}^{G}\;\Delta b_{\mathrm{clk}}^{N}\right]}_{\ell }^{T}$ vectors for all *ℓ* noted after Equation (16). ${\underline{\eta }}_{1}$ is measurement error. The diagonal terms of its covariance matrix, Ψ, are unity. ${\tilde{S}}^{\prime }{{\tilde{S}}^{\prime }}^{T}$ present on either side of Θ in Equation (16) removes $H^{*}\Delta {\underline{\varkappa }}_{p}$ from $\Delta {\underline{\tilde{\rho }}}^{*}$, ensuring that the fault of only *ℓ*th epoch is injected through $\Delta {\underline{\rho }}_{\ell }^{*}$. This enables formation of more than one test statistic, each with a certain number of epochs, as before. If a term from one epoch also injects faults of other epochs, formation of separate test statistics is difficult from the point of view of the corresponding FMS computation, as discussed earlier. This would be the case if ${\tilde{S}}^{\prime }{{\tilde{S}}^{\prime }}^{T}$ were omitted from Equation (16). Grouping ${{\tilde{S}}^{\prime }}^{T}\Delta {\underline{\tilde{\rho }}}^{*}$ as $\Delta {\underline{\tilde{\rho }}}^{\prime }$, Equation (16) becomes

(18) $${\left(\alpha_{j}^{\prime }\right)}^{2}={\Delta {\underline{\tilde{\rho }}}^{\prime }}^{T}\left({{\tilde{S}}^{\prime }}^{T}\Theta {\tilde{S}}^{\prime }\right)\Delta {\underline{\tilde{\rho }}}^{\prime }$$

where ${{\tilde{S}}^{\prime }}^{T}\Theta {\tilde{S}}^{\prime }$ (or $\Theta_{1}$) has $\sum_{\ell =p_{j}}^{m_{j}}\left(n-n_{\mathrm{null}}\right)$ rows/columns. The covariance matrix $\Psi_{1}$ of error in $\Delta {\underline{\tilde{\rho }}}^{\prime }$, ${\underline{\eta }}_{2}$ is given by

(19) $$\Psi_{1}=E\left({\underline{\eta }}_{2}{\underline{\eta }}_{2}^{T}\right)={{\tilde{S}}^{\prime }}^{T}\Psi {\tilde{S}}^{\prime }$$

where *E* is the expectation operator. Ψ is the covariance matrix of measurement error in $\Delta {\underline{\tilde{\rho }}}^{*}$, ${\underline{\eta }}_{1}$. Measurement error comprises broadcast ephemeris and satellite clock error, unmodeled tropospheric delay, multipath and noise. It is assumed that the pseudorange measurement error of each visible satellite is a first order GM process with time constant 1/β and of zero mean under no faults.

Since time correlated errors are not estimated by the SKF, they remain unchanged in the pseudorange innovation terms. In this context, it should be noted that if time correlated errors were estimated by augmenting states in EKF, it would not be possible to formulate the RAIM algorithm described here. This is because if the augmented states for time correlated errors (one for each pseudorange innovation) were included in the WLS estimation of fault detection method, the resulting system would be under-determined. If the augmented states of EKF were not included in WLS estimation, faults of other epochs would be injected through the pseudorange innovation term of an epoch. This is because of possible wrong estimation of augmented states in EKF in the presence of a fault. This would complicate the calculation of FMS, and a separate FMS for each test statistic would not be possible. The reason for preferring a WLS-based fault detection approach was discussed in Section 3.2 and before Equation (18). Therefore, implementation of the SKF is essential to the formulation of the range-based KF RAIM algorithm presented in this paper. SKF is computationally efficient, and provides reasonably good performance as compared to that of the EKF that estimates additional states for time correlated errors [43]. Next, Ψ and Θ are formulated for the first test statistic with *M* epochs (see paragraph before Equation (3) for the definition of *M*).

#### 5.1.1. Formulation of Ψ for First Test Statistic

First, Ψ is determined. Expanding ${\underline{\eta }}_{1}$

(20) $$\begin{array}{c} {\underline{\eta }}_{1}={\left[{\underline{\eta }}_{1}^{\left(1\right)}\left(1\right)\cdots {\underline{\eta }}_{1}^{\left(1\right)}\left(n\right)\;{\underline{\eta }}_{1}^{\left(2\right)}\left(1\right)\cdots {\underline{\eta }}_{1}^{\left(2\right)}\left(n\right)\cdots {\underline{\eta }}_{1}^{\left(\ell \right)}\left(1\right)\cdots {\underline{\eta }}_{1}^{\left(\ell \right)}\left(n\right)\cdots {\underline{\eta }}_{1}^{\left(M\right)}\left(1\right)\cdots {\underline{\eta }}_{1}^{\left(M\right)}\left(n\right)\right]}^{T} \end{array}$$

where the superscript within parentheses denotes time instants, and the argument indicates pseudorange measurement index (or satellite index) at a time instant. Total number of visible satellites, *n* can vary across epochs. By definition, Ψ (=$E\left({\underline{\eta }}_{1}{\underline{\eta }}_{1}^{T}\right)$) is

(21) $$\begin{array}{cc} \Psi =\left[\begin{array}{cccc} E\left({\left({\underline{\eta }}_{1}^{\left(1\right)}\left(1\right)\right)}^{2}\right) & {\left[0\right]}_{1\times (n-1)} & E\left({\underline{\eta }}_{1}^{\left(1\right)}\left(1\right){\underline{\eta }}_{1}^{\left(2\right)}\left(1\right)\right) & \cdots \\ 0 & E\left({\left({\underline{\eta }}_{1}^{\left(1\right)}\left(2\right)\right)}^{2}\right) & {\left[0\right]}_{1\times (n-1)} & \cdots \\ {\left[0\right]}_{(n-2)\times 1} & \cdots & \; & \\ E\left({\underline{\eta }}_{1}^{\left(2\right)}\left(1\right){\underline{\eta }}_{1}^{\left(1\right)}\left(1\right)\right) & \cdots & \\ & \vdots & \end{array}\right] & \end{array}$$

where [0] is a matrix with zero elements. Its dimension is shown in the subscript. For any satellite *i*, at epochs *ℓ* and ℓ+q, with *q* > 0

(22) $$E\left({\underline{\eta }}_{1}^{\left(\ell \right)}\left(i\right){\underline{\eta }}_{1}^{\left(\ell +q\right)}\left(i\right)\right)=\frac{E\left({\underline{\eta }}^{\left(\ell \right)}\left(i\right){\underline{\eta }}^{\left(\ell +q\right)}\left(i\right)\right)}{\sigma^{\left(\ell \right)}\left(i\right)\sigma^{\left(\ell +q\right)}\left(i\right)}$$

where $\underline{\eta }$ is measurement error in $\Delta \underline{\tilde{\rho }}$ (which stacks relevant $\Delta \underline{\rho_{\ell }}$ terms), and σ denotes standard deviation of its individual element. Using GM model, one can write

(23) $$\begin{array}{cc} {\underline{\eta }}^{\left(\ell +1\right)}\left(i\right) & =\exp^{\left(-\beta \Delta t\right)}{\underline{\eta }}^{\left(\ell \right)}\left(i\right)+\zeta_{i}^{\left(\ell \right)} \end{array}$$

(24) $$\begin{array}{cc} E\left({\underline{\eta }}_{1}^{\left(\ell \right)}\left(i\right){\underline{\eta }}_{1}^{\left(\ell +q\right)}\left(i\right)\right)= & {\left(\exp^{\left(-\beta \Delta t\right)}\right)}^{q}\frac{E\left({\left({\underline{\eta }}^{\left(\ell \right)}\left(i\right)\right)}^{2}\right)}{\sigma^{\left(\ell \right)}\left(i\right)\sigma^{\left(\ell +q\right)}\left(i\right)} \end{array}$$

where Δt = measurement update interval = 1 s. $\zeta_{i}^{\left(\ell \right)}$ is white noise sample for satellite *i* and uncorrelated with ${\underline{\eta }}^{\left(\ell \right)}\left(i\right)$. Simplifying,

(25) $$E\left({\underline{\eta }}_{1}^{\left(\ell \right)}\left(i\right){\underline{\eta }}_{1}^{\left(\ell +q\right)}\left(i\right)\right)={\left(\exp^{\left(-\beta \Delta t\right)}\right)}^{q}\frac{\sigma^{\left(\ell \right)}\left(i\right)}{\sigma^{\left(\ell +q\right)}\left(i\right)}$$

Substituting this into Equation (21)

(26) $$\begin{array}{c} \Psi =\left[\begin{array}{cccccc} 1 & {\left[0\right]}_{1\times (n-1)} & \exp^{\left(-\beta \Delta t\right)}\frac{\sigma^{\left(1\right)}\left(1\right)}{\sigma^{\left(2\right)}\left(1\right)} & {\left[0\right]}_{1\times (n-1)} & \cdots & 0\\ & & \vdots & & & \vdots \\ & & \cdots & & & \exp^{\left(-\beta \Delta t\right)}\frac{\sigma^{\left(M-1\right)}\left(n\right)}{\sigma^{\left(M\right)}\left(n\right)}\\ & & \vdots & & & {\left[0\right]}_{(n-1)\times 1}\\ 0 & \cdots & & \exp^{\left(-\beta \Delta t\right)}\frac{\sigma^{\left(M-1\right)}\left(n\right)}{\sigma^{\left(M\right)}\left(n\right)} & {\left[0\right]}_{1\times (n-1)} & 1 \end{array}\right] \end{array}$$

The term $\frac{\sigma^{\left(\ell \right)}\left(i\right)}{\sigma^{\left(\ell +q\right)}\left(i\right)}$ can be written as

(27) $$\frac{\sigma^{\left(\ell \right)}\left(i\right)}{\sigma^{\left(\ell +q\right)}\left(i\right)}=\frac{\sigma^{\left(\ell \right)}\left(i\right)}{\sigma^{\left(\ell +1\right)}\left(i\right)}\frac{\sigma^{\left(\ell +1\right)}\left(i\right)}{\sigma^{\left(\ell +2\right)}\left(i\right)}\frac{\sigma^{\left(\ell +2\right)}\left(i\right)\cdots }{\sigma^{\left(\ell +3\right)}\left(i\right)\cdots }\frac{\sigma^{\left(\ell +q-1\right)}\left(i\right)}{\sigma^{\left(\ell +q\right)}\left(i\right)}$$

**Figure 2:**
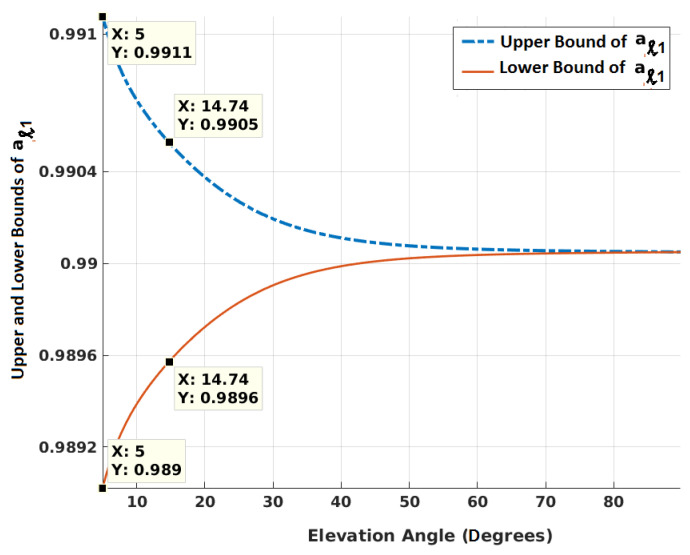
Variation of upper and lower bounds of $\exp^{\left(-\beta \Delta t\right)}\times \frac{\sigma^{\left(\ell \right)}\left(i\right)}{\sigma^{\left(\ell +1\right)}\left(i\right)}$ for GPS satellites for different elevation angles at epoch *ℓ*, and Δt = 1 s.

It is a product of *q* ratios. Each ratio will have an accompanying $\exp^{\left(-\beta \Delta t\right)}$ when ${\left(\exp^{\left(-\beta \Delta t\right)}\right)}^{q}$ is multiplied with the product. Using Equations (9)–(11), Figure 2 shows how the upper and lower bounds of $\exp^{\left(-\beta \Delta t\right)}\times \frac{\sigma^{\left(\ell \right)}\left(i\right)}{\sigma^{\left(\ell +1\right)}\left(i\right)}$ (=$a_{\ell 1}$) vary for GPS satellites with elevation angles $\theta^{\left(\ell \right)}$ at epoch *ℓ*. It is evident from the equations that maximum change in elevation angle between two successive epochs would maximize $a_{\ell 1}$, which is also higher when $\theta^{\left(\ell +1\right)}$ > $\theta^{\left(\ell \right)}$. The maximum change in elevation angle between two epochs of 1 s interval is calculated in radians as follows.

(28) $$\theta^{\left(\ell +1\right)}-\theta^{\left(\ell \right)}=\frac{v_{\max}^{s}+v_{\max}^{u}}{r_{\min}^{su}}$$

where $v_{\max}^{s}$ is the maximum satellite speed, $v_{\max}^{u}$ is the maximum user speed (assumed Mach 1) and $r_{\min}^{su}$ is the minimum range between a satellite and user at an elevation angle. The maximum altitude of the user is 15 km. When $\theta^{\left(\ell +1\right)}$ < $\theta^{\left(\ell \right)}$, following the same procedure, lower bounds of $a_{\ell 1}$ are obtained. The term $a_{\ell 1}$ calculated for GPS is found to be greater and less than that of NavIC with respect to upper and lower bounds, respectively. This is because NavIC satellites are located in geosynchronous orbits. As shown in the figure, the largest value of $a_{\ell 1}$, $a_{\max}$ is 0.9911 at 5${}^{\circ }$ mask angle. The lowest value of $a_{\ell 1}$, $a_{\min}$ is 0.9890. A matrix $\Psi_{\max}$ is modeled as

(29) $$\begin{array}{cc} \Psi_{\max}= & \left[\begin{array}{cccccc} 1 & {\left[0\right]}_{1\times (n-1)} & a_{\max} & {\left[0\right]}_{1\times (n-1)} & \cdots & 0\\ 0 & 1 & {\left[0\right]}_{1\times (n-1)} & a_{\max} & & \cdots \\ {0]}_{(n-2)\times 1} & & & & & \\ a_{\max} & & \cdots & & & \\ {0]}_{(n-1)\times 1} & & & & & \vdots \\ & & \vdots & & & \\ & & \cdots & & & a_{\max}\\ & & \vdots & & & {\left[0\right]}_{(n-1)\times 1}\\ 0 & \cdots & & a_{\max} & {\left[0\right]}_{1\times (n-1)} & 1 \end{array}\right] \end{array}$$

The determinant of $\Psi_{\max}$ can be easily calculated by the product of the determinants of all sub-matrices—each formed by grouping together the non-zero elements for a particular satellite. This is because measurements are assumed uncorrelated across satellites. Thus, with some rearrangements of rows and columns, it can be shown that $\Psi_{\max}$ is a block diagonal matrix formed by these sub-matrices. Each sub-matrix is of the form $\Psi_{\mathrm{sub},}^{i}\;\max$ = $\left[\begin{array}{ccccc} 1 & a_{\max} & a_{\max}^{2} & a_{\max}^{3} & \cdots \\ a_{\max} & 1 & a_{\max} & a_{\max}^{2} & \cdots \\ a_{\max}^{2} & a_{\max} & 1 & a_{\max} & \cdots \\ & & \vdots & \end{array}\right]$, where *i* is satellite index. Its dimension is determined by the number of time instants when the satellite is visible within *M* epochs. If it is visible throughout, then the determinant of the sub-matrix is ${\left(1-a_{\max}^{2}\right)}^{\left(M-1\right)}$. It is greater than zero, and approaches zero as *M* increases, but theoretically is not equal to zero for a finite M. All leading principal minors are also positive. Thus, each sub-matrix is positive definite, and has all positive eigenvalues. Combining all sub-matrices, one can say that $\Psi_{\max}$ is positive definite.

In case of Ψ, following Equations (26) and (27), the sub-matrix is of the form $\Psi_{\mathrm{sub}}^{i}$ = $\left[\begin{array}{ccccc} 1 & a_{11} & a_{11}a_{21} & a_{11}a_{21}a_{31} & \cdots \\ a_{11} & 1 & a_{21} & a_{21}a_{31} & \cdots \\ a_{11}a_{21} & a_{21} & 1 & a_{31} & \cdots \\ & & \vdots & \end{array}\right]$, where $a_{11}$, $a_{21}$, and $a_{31}$ are values of $a_{\ell 1}$ at epochs $t_{\ell }$ = $t_{1}$, $t_{2}$ and $t_{3}$, respectively. Its determinant is $\prod_{\ell =1}^{\left(M-1\right)}\left(1-a_{\ell 1}^{2}\right)$. Using the same argument as earlier, Ψ is positive definite. Since ${\tilde{S}}^{\prime }$ has full column rank, $\Psi_{1}$ (=${{\tilde{S}}^{\prime }}^{T}\Psi {\tilde{S}}^{\prime }$) is also positive definite. This implies that there exists a matrix $\Psi_{2}$ such that $\Psi_{1}$ = $\Psi_{2}{\Psi_{2}}^{T}$.

**Figure 3:**
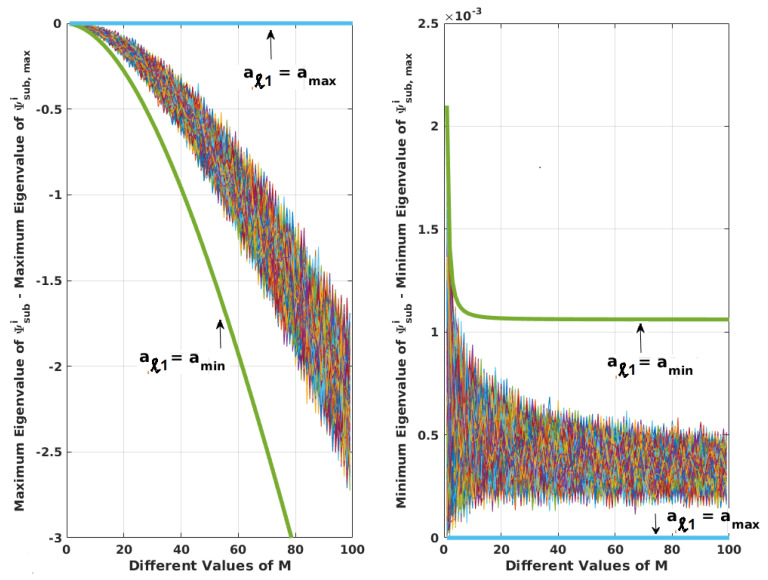
Comparison of maximum and minimum eigenvalues of $\Psi_{\mathrm{sub}}^{i}$ and $\Psi_{\mathrm{sub},\;\max}^{i}$ for different *M*. There are $10^{4}$ values for each *M*. Upper and lower limits are shown, ∀$a_{\ell 1}$ = $a_{min}$ and $a_{\ell 1}$ = $a_{max}$. This figure can be regenerated with the Matlab program, matlab_prog_figures_3_A1.m provided in the Supplementary Materials.

For $a_{\ell 1}$ between $\left[a_{min},\;a_{max}\right]$, it is evident from the mathematical expressions that the determinant of $\Psi_{\mathrm{sub},\;\max}^{i}$, $|\Psi_{\mathrm{sub},\;\max}^{i}|$ ≤ $|\Psi_{\mathrm{sub}}^{i}|$. $\Psi_{\mathrm{sub},\;\max}^{i}$ can also be written as $\Psi_{\mathrm{sub},\;\max}^{i}$ = $\Psi_{\mathrm{sub}}^{i}$ + $\Delta \Psi_{\mathrm{sub}}^{i}$, where all diagonal terms of $\Delta \Psi_{\mathrm{sub}}^{i}$ are zero, and off-diagonal terms are positive ∀$a_{\ell 1}$ ≠ $a_{max}$. This is equivalent to perturbing $\Psi_{\mathrm{sub}}^{i}$ so as to make it move closer to singularity. Hence, the condition number of $\Psi_{\mathrm{sub},\;\max}^{i}$ (ratio of maximum to minimum eigenvalue for a symmetric matrix) would be greater. Figure 3 justifies this for different *M* by generating $\Psi_{\mathrm{sub}}^{i}$$10^{4}$ times for each *M* with uniformly random $a_{\ell 1}$ between $\left[a_{min},\;a_{max}\right]$, and comparing its eigenvalues with those of $\Psi_{\mathrm{sub},\;\max}^{i}$. From the figure, it can be concluded that the minimum (or maximum) eigenvalue of $\Psi_{\mathrm{sub},\;\max}^{i}$ is less (or greater) than the respective eigenvalue of $\Psi_{\mathrm{sub}}^{i}$, when $a_{\ell 1}$s are not equal to $a_{max}$. Thus, the maximum and minimum eigenvalues of Ψ can be related to those of $\Psi_{\max}$ as

(30) $$\lambda_{\Psi }^{\max}\leq \lambda_{\Psi ,\;max}^{\max},\;\lambda_{\Psi }^{\min}\geq \lambda_{\Psi ,\;max}^{\min}$$

where $\lambda_{J}$ represents an eigenvalue of a matrix J and its superscript shows if it is maximum or minimum. Equation (30) will be useful while discussing the second test statistic.

#### 5.1.2. Formulation of Θ for First Test Statistic

Next, Θ is determined so that the fault detection threshold of $\alpha_{j}^{\prime }$ can be obtained. For ease of understanding, ${\left(\alpha_{j}^{\prime }\right)}^{2}$ and the mathematical model of $\Delta {\underline{\tilde{\rho }}}^{*}$ are repeated here.

(31) $$\begin{array}{cc} {\left(\alpha_{j}^{\prime }\right)}^{2} & ={\Delta {\underline{\tilde{\rho }}}^{*}}^{T}{\tilde{S}}^{\prime }{{\tilde{S}}^{\prime }}^{T}\Theta {\tilde{S}}^{\prime }{{\tilde{S}}^{\prime }}^{T}\Delta {\underline{\tilde{\rho }}}^{*}={\Delta {\underline{\tilde{\rho }}}^{\prime }}^{T}\left({{\tilde{S}}^{\prime }}^{T}\Theta {\tilde{S}}^{\prime }\right)\Delta {\underline{\tilde{\rho }}}^{\prime } \end{array}$$

(32) $$\begin{array}{cc} \Delta {\underline{\tilde{\rho }}}^{*} & =H^{*}\Delta {\underline{\varkappa }}_{p}+{\underline{\eta }}_{1} \end{array}$$

Since ${\tilde{S}}^{\prime }{{\tilde{S}}^{\prime }}^{T}$ removes $H^{*}\Delta {\underline{\varkappa }}_{p}$ from $\Delta {\underline{\tilde{\rho }}}^{*}$, the distribution of ${\left(\alpha_{j}^{\prime }\right)}^{2}$ is determined by the measurement error ${\underline{\eta }}_{1}$. Hence, only ${\underline{\eta }}_{1}$ of $\Delta {\underline{\tilde{\rho }}}^{*}$ will be considered in subsequent derivations. Using measurement error, Equation (31) results in

(33) $${{\underline{\eta }}_{2}}^{T}\left({{\tilde{S}}^{\prime }}^{T}\Theta {\tilde{S}}^{\prime }\right){\underline{\eta }}_{2}$$

Replacing ${\underline{\eta }}_{2}$ with $\Psi_{2}{\underline{\eta }}_{3}$, where ${\underline{\eta }}_{3}$ has unit-variance independent elements (since $E\left({\underline{\eta }}_{2}{\underline{\eta }}_{2}^{T}\right)=\Psi_{1}$ = $\Psi_{2}\Psi_{2}^{T}$)

(34) $${{\underline{\eta }}_{3}}^{T}{\Psi_{2}}^{T}\left({{\tilde{S}}^{\prime }}^{T}\Theta {\tilde{S}}^{\prime }\right)\Psi_{2}{\underline{\eta }}_{3}$$

Putting $\Theta_{2}$ in place of ${\Psi_{2}}^{T}\left({{\tilde{S}}^{\prime }}^{T}\Theta {\tilde{S}}^{\prime }\right)\Psi_{2}$

(35) $${{\underline{\eta }}_{3}}^{T}\Theta_{2}{\underline{\eta }}_{3}$$

Reference [49] proves that the fault detection threshold of the preceding term can be obtained from the Chi square distribution if the maximum eigenvalue of $\Theta_{2}$ is less than or equal to one. Θ is determined next to satisfy this. If $\lambda_{\Theta ,\;2}$ is an eigenvalue of $\Theta_{2}={\Psi_{2}}^{T}\left({{\tilde{S}}^{\prime }}^{T}\Theta {\tilde{S}}^{\prime }\right)\Psi_{2}$, and $\underline{v}$ is the corresponding eigenvector, then

(36) $${\Psi_{2}}^{T}\left({{\tilde{S}}^{\prime }}^{T}\Theta {\tilde{S}}^{\prime }\right)\Psi_{2}\underline{v}=\lambda_{\Theta ,\;2}\underline{v}$$

Multiplying $\Psi_{2}$ on either side and denoting $\Psi_{2}\underline{v}$ as ${\underline{v}}_{1}$

$$\Psi_{2}{\Psi_{2}}^{T}\left({{\tilde{S}}^{\prime }}^{T}\Theta {\tilde{S}}^{\prime }\right){\underline{v}}_{1}=\lambda_{\Theta ,\;2}{\underline{v}}_{1}$$

Replacing $\Psi_{2}{\Psi_{2}}^{T}$ with $\Psi_{1}$ and $\left({{\tilde{S}}^{\prime }}^{T}\Theta {\tilde{S}}^{\prime }\right)$ with $\Theta_{1}$, it is clear that the eigenvalues of $\Psi_{1}\Theta_{1}$ (denoted by Ω later) are identical with those of $\Theta_{2}$. Therefore, in subsequent derivations, the eigenvalues of Ω will be considered.

The following form of Θ is proposed.

(37) $$\Theta ={\left(cI+\Psi \right)}^{-1}$$

where *c* is a positive scale factor, which is determined such that the maximum eigenvalue of Ω is less than or equal to unity. From the preceding equation it is evident that for a positive value of *c*, Θ is a positive definite matrix. Since both ${\tilde{S}}^{\prime }$ and $\Psi_{2}$ have full column rank, ${\Psi_{2}}^{T}\left({{\tilde{S}}^{\prime }}^{T}\Theta {\tilde{S}}^{\prime }\right)\Psi_{2}$ or Ω (=$\Psi_{1}\Theta_{1}$) have all positive eigenvalues. Next, it will be proved that there is a value of *c*, for which the maximum eigenvalue of Ω, $\lambda_{\Omega }^{\max}$ is less than unity. If ${\left||.|\right|}_{2}$ is the 2-norm of a matrix

(38) $$\lambda_{\Omega }^{\max}<||\Psi_{1}\Theta_{1}{\left|\right|}_{2}\leq \left|\right|\Psi_{1}{\left|\right|}_{2}\left|\right|\Theta_{1}{\left|\right|}_{2}\leq {\left||\Psi |\right|}_{2}{\left||\Theta |\right|}_{2}$$

It should be noted that $\left|\right|{\tilde{S}}^{\prime }{\left|\right|}_{2}$ is unity. Since both Ψ and Θ are symmetric, their maximum eigenvalues are equal to respective 2-norms. Hence, $\lambda_{\Psi }^{\max}$ = ${\left||\Psi |\right|}_{2}$ and $\lambda_{\Theta }^{\max}$ = ${\left||\Theta |\right|}_{2}$. An expression of $\lambda_{\Theta }^{\max}$ is derived next. If $\lambda_{J}$ represents an eigenvalue of a matrix J, then it can be easily shown that

(39) $$\lambda_{\left(cI+\Psi \right)}=c+\lambda_{\Psi }$$

For matrix $\Theta ={\left(cI+\Psi \right)}^{-1}$, $\lambda_{\Theta }$ = $1/(c+\lambda_{\Psi })$. Thus, the maximum eigenvalue of Θ, $\lambda_{\Theta }^{\max}$ = $1/(c+\lambda_{\Psi }^{\min})$, where $\lambda_{\Psi }^{\min}$ is the minimum eigenvalue of Ψ. Therefore,

(40) $${\left||\Psi |\right|}_{2}{\left||\Theta |\right|}_{2}=\frac{\lambda_{\Psi }^{\max}}{\left(c+\lambda_{\Psi }^{\min}\right)}$$

If *c* is chosen equal to $\left(\lambda_{\Psi }^{\max}-\lambda_{\Psi }^{\min}\right)$, $\lambda_{\Omega }^{\max}$ is less than unity.

However, the above *c* results in a large FMS corresponding to the first test statistic. Since the FMS of the first test statistic significantly contributes to determining the PL, it should be reduced by selecting a small *c*. A computationally efficient algorithm to adaptively find Θ for $\alpha_{1}^{\prime }$ at every epoch is provided in Appendix C. Ψ is recursively computed at every $t_{k}$ with $\Psi_{\mathrm{sub}}^{i}$ for all satellites. It should be noted that even after a satellite sets, its measurement innovations are used to form test statistics as long as they are within *N* epochs from the current epoch.

#### 5.1.3. Formulation of Θ for Second Test Statistic

Ψ and $\Psi_{\max}$ for the second test statistic, $\alpha_{2}$ can be formulated the same way as before, but for (N−M) epochs. As the FMS corresponding to $\alpha_{2}$ is generally small, Θ for it is calculated as

(41) $${\left(cI+\Psi_{\max}\right)}^{-1}$$

where *c* is provided after Equation (40), but calculated with the minimum and maximum eigenvalues of $\Psi_{\max}$. In this case,

(42) $$\lambda_{\Omega }^{\max}{<||\Psi ||}_{2}{\left||\Theta |\right|}_{2}=\frac{\lambda_{\Psi }^{\max}}{\left(c+\lambda_{\Psi ,\;\max}^{\min}\right)}$$

Substituting for *c* and using Equation (30), it is evident that $\lambda_{\Omega }^{\max}$< 1. Θ can be pre-determined for various (N−M). Since $\Psi_{\max}$ can be put in the form of a block diagonal matrix, it is sufficient to pre-calculate the inverse of $\left(cI+\Psi_{\mathrm{sub},\;\;\max}^{i}\right)$ for various (N−M). $\Psi_{\mathrm{sub},\;\;\max}^{i}$ and its pre-calculated inverse are loaded into the program. With this, Θ is appropriately formed at every $t_{k}$ for all visible satellites over epochs $t_{k-N+1}$ to $t_{k-M}$. If a satellite *q* is visible for a shorter time during this interval, then part of Θ corresponding to $\Psi_{\mathrm{sub},\;\;\max}^{q}$ is recursively calculated starting from ${\left(cI+\Psi_{\mathrm{sub},\;\;\max}^{i}\right)}^{-1}$, following [50]. Satellite *i* is assumed to be visible for the longest duration over (N−M) epochs. Hence, $\lambda_{\Psi ,\;\max}^{\min\;(\mathrm{or}\;\max)}$ = minimum (or maximum) eigenvalue of $\Psi_{\mathrm{sub},\;\;\max}^{i}$. Next, the final form of test statistics and their thresholds are discussed.

#### 5.1.4. Test Statistics and Thresholds

In this work, the third test formulated with all discarded terms of epochs before $t_{k-N+1}$ is not performed. The following subsection computes a bound on the contributions of the discarded terms to mean position error without requiring an FMS (or a corresponding test). Thus, Equation (3) is modified as

(43) $$\begin{array}{cc} \alpha_{j}^{2} & ={\Delta {\underline{\tilde{\rho }}}^{*}}^{T}{\tilde{S}}^{\prime }{{\tilde{S}}^{\prime }}^{T}\Theta {\tilde{S}}^{\prime }{{\tilde{S}}^{\prime }}^{T}\Delta {\underline{\tilde{\rho }}}^{*}+\sum_{\ell =p_{j}}^{m_{j}}\left(\Delta {\underline{\dot{\rho }}}_{\ell }^{T}{W_{rr,\;\ell }}^{-1}\left(I-G_{\ell }^{rr}\right)\Delta {\underline{\dot{\rho }}}_{\ell }\right) \end{array}$$

where *j* = 1, 2. Since ${\tilde{S}}^{\prime }{{\tilde{S}}^{\prime }}^{T}$ is a block diagonal matrix with each block from an epoch, elements of the vector ${\tilde{S}}^{\prime }{{\tilde{S}}^{\prime }}^{T}\Delta {\underline{\tilde{\rho }}}^{*}$ corresponding to an epoch are calculated at that epoch and then stacked along with those of other epochs. This eliminates unnecessarily large matrix multiplications.

With the designed Ψ and Θ, fault detection thresholds, $T_{th,\;1}^{2}$ for $\alpha_{1}^{2}$ and $T_{th,\;2}^{2}$ for $\alpha_{2}^{2}$, can be determined from the Chi square distribution with probability $P_{FA}/2$ and DOF = $\sum_{\ell =p_{j}}^{m_{j}}2\left(n-n_{\mathrm{null}}\right)$, where $n_{\mathrm{null}}$ is the dimension of the null space of $\left(I-G_{\ell }^{*}\right)$. The number two is multiplied for the second term of Equation (43). If either $\alpha_{1}$ exceeds $T_{th,\;1}$ or $\alpha_{2}$ exceeds $T_{th,\;2}$ or both happen, a fault is declared. Calculation of the mean position error bounds is detailed next.

### 5.2. Mean Position Error Bounds under Faults

Changes to mean position error bound computation mentioned in Section 3.2 are explained in this subsection. Since the contribution of nonlinear measurement model (see Equations (6) and (7) and discussion after them) was found to be very small, it is not considered in this paper. Next, the three terms under the summation of Equation (7) are discussed for the vertical position error (VPE). Terms for horizontal position error (HPE) are changed accordingly. The mean position error, ${\underline{\mu }}_{k}^{\prime }$ at time $t_{k}$ in the north-east-down (NED) frame is

(44) $$\begin{array}{cc} {\underline{\mu }}_{k}^{\prime } & =-\underset{\mathrm{discarded}\;\mathrm{terms}}{\underset{︸}{\sum_{j=N-1}^{k-(m+1)}B_{k-j}^{\prime }{\underline{f}}_{k-m-j}}}-\underset{N\;\mathrm{terms}\;\mathrm{corresponding}\;\mathrm{to}\;\alpha_{1}\;\&\;\alpha_{2}}{\underset{︸}{\left(\sum_{j=0}^{N-2}B_{k-j}^{\prime }{\underline{f}}_{k-m-j}+K_{s,\;k}^{\prime }{\underline{f}}_{\;k-m+1}\right)}} \end{array}$$

where ${\underline{\mu }}_{k}^{\prime }$ = $C_{e}^{n}{\underline{\mu }}_{k}\left(1:2:5\right)$, ${\underline{\mu }}_{k}$ is the mean error of all estimated states and (1:2:5) represents elements for the position states, $B_{k-j}^{\prime }$ = $C_{e}^{n}B_{k-j}\left(1:2:5,:\right)$, $B_{k-j}$ = $\left[\prod_{\ell =k}^{k-j}A_{\ell }\right]K_{s,\;k-j-1}$, $A_{\ell }\;=\;\left(I-K_{s,\;\ell }C_{s,\;\ell }\right)F_{s,\;\ell -1}$. $K_{s}$, $F_{s}$ and $C_{s}$ are Kalman gain, state transition and measurement model matrices of estimated SKF states, respectively. They are defined in Appendix B. $K_{s,\;k}^{\prime }$ = $C_{e}^{n}K_{s,\;k}\left(1:2:5,:\right)$, where J(1:2:5,:) has rows one, three and five of matrix J. $C_{e}^{n}$ is the ECEF to NED frame coordinate transformation matrix. *N* terms corresponding to two test statistics and discarded terms are separately grouped with under braces. Fault is assumed to have started at an epoch *m* and denoted by vector ${\underline{f}}_{k-m+1}$ at $t_{k}$. Up to two independent satellite faults are considered. As noted earlier, three or more faults are not monitored. Thus, for any two satellites $s_{1}$ and $s_{2}$, ${\underline{f}}_{k-m+1}\left(s_{1}\right)=b_{1,\;k-m+1}$, ${\underline{f}}_{k-m+1}\left(s_{1}+n\right)={\dot{b}}_{1,\;k-m+1}$, ${\underline{f}}_{k-m+1}\left(s_{2}\right)=b_{2,\;k-m+1}$, ${\underline{f}}_{k-m+1}\left(s_{2}+n\right)={\dot{b}}_{2,\;k-m+1}$ and all other elements of ${\underline{f}}_{k-m+1}$ are zero. For SF cases, only $s_{1}th$ satellite is assumed faulty. The vertical component of mean position error bound for $\alpha_{1}$ under fault mode *i*, $VPE_{1,\;i}^{U}$ is calculated as follows.

#### 5.2.1. $VPE_{1,\;i}^{U}$

**Figure 4:**
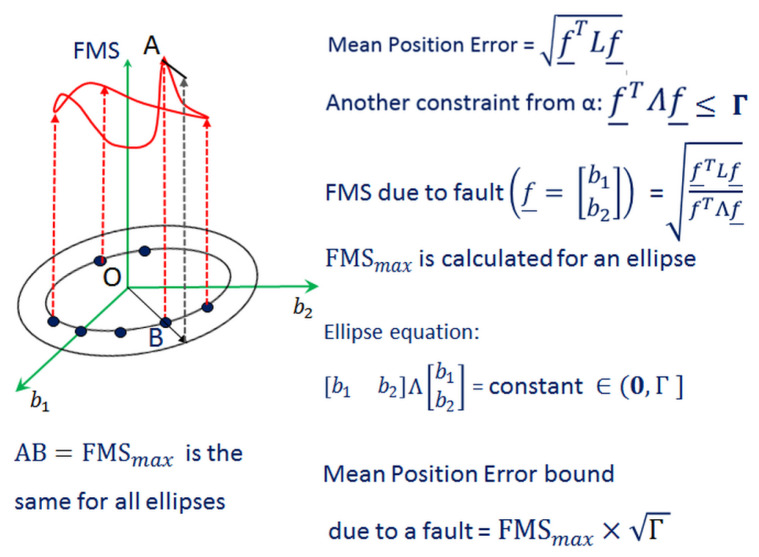
Illustration of FMS for a hypothetical two dimensional fault vector. The maximum FMS (FMS${}_{max}$) for each ellipse is the same. The mean position error bound is the product of maximum FMS and $\sqrt{\Gamma }$.

For a fault mode *i* (=$1,\ldots ,\;N_{fault}$), square of the maximum FMS [20] corresponding to the first test statistic $\alpha_{1}$ with terms of *M* time epochs from *k* to k−M+1 is given by

(45) $$(\mathrm{maximum}\;\;{\mathrm{FMS})}_{\alpha 1,\;i}^{2}=\max_{{\underline{f}}_{w}^{T}\Lambda {\underline{f}}_{w}=\Gamma }\frac{{\underline{f}}_{w}^{T}L{\underline{f}}_{w}}{{\underline{f}}_{w}^{T}\Lambda {\underline{f}}_{w}}$$

where ${\underline{f}}_{w}$, the worst-case fault vector, maximizes the FMS. A pictorial depiction of FMS is provided in Figure 4.

For a DF mode in satellites $s_{1}$ and $s_{2}$, L is

(46) $$\begin{array}{cc} L=[B_{k-(M-2)}^{\prime }\left(3,\;s_{1}\right)\;B_{k-(M-2)}^{\prime }\left(3,\;s_{1}+n\right)\;B_{k-(M-2)}^{\prime }\left(3,\;s_{2}\right)\;\\ B_{k-(M-2)}^{\prime }\left(3,\;s_{2}+n\right)\;\ldots \;B_{k}^{\prime }\left(3,\;s_{1}\right)\;B_{k}^{\prime }\left(3,\;s_{1}+n\right)\;\\ B_{k}^{\prime }\left(3,\;s_{2}\right)\;B_{k}^{\prime }\left(3,\;s_{2}+n\right)\;K_{s,\;k}^{\prime }\left(3,\;s_{1}\right)\;K_{s,\;k}^{\prime }\left(3,\;s_{1}+n\right)\;\\ K_{s,\;k}^{\prime }\left(3,\;s_{2}\right)\;K_{s,\;k}^{\prime }\left(3,\;s_{2}+n\right){]}^{T}\\ \times [B_{k-(M-2)}^{\prime }\left(3,\;s_{1}\right)\;B_{k-(M-2)}^{\prime }\left(3,\;s_{1}+n\right)\;B_{k-(M-2)}^{\prime }\left(3,\;s_{2}\right)\;\\ B_{k-(M-2)}^{\prime }\left(3,\;s_{2}+n\right)\;\ldots \;B_{k}^{\prime }\left(3,\;s_{1}\right)\;B_{k}^{\prime }\left(3,\;s_{1}+n\right)\;\\ B_{k}^{\prime }\left(3,\;s_{2}\right)\;B_{k}^{\prime }\left(3,\;s_{2}+n\right)\;K_{s,\;k}^{\prime }\left(3,\;s_{1}\right)\;K_{s,\;k}^{\prime }\left(3,\;s_{1}+n\right)\;\\ K_{s,\;k}^{\prime }\left(3,\;s_{2}\right)\;K_{s,\;k}^{\prime }\left(3,\;s_{2}+n\right)] & \end{array}$$

where ‘×’ represents matrix multiplication operation. Although it is not evident, the number of visible satellites, *n* can vary across epochs. ${\underline{f}}_{w}$ has the corresponding $b_{1}$, ${\dot{b}}_{1}$, $b_{2}$ and ${\dot{b}}_{2}$ terms from Equation (44). For a SF mode, elements for only satellite $s_{1}$ are considered.

It should be noted that for the HPE, first and second rows of $B^{\prime }$ and $K_{s}^{\prime }$ are used to form L. ${\underline{f}}_{w}$ is composed of the corresponding $b_{1}$, ${\dot{b}}_{1}$, $b_{2}$ and ${\dot{b}}_{2}$ terms. Λ of Equation (45) is given as

(47) $$\begin{array}{cc} \Lambda = & \left[\begin{array}{cc} \Theta_{3} & \left[0\right]\\ \left[0\right] & \Theta_{4} \end{array}\right] \end{array}$$

where $\Theta_{3}$ is formed with terms of ${\tilde{W}}^{-1/2}{\tilde{S}}^{\prime }{{\tilde{S}}^{\prime }}^{T}\Theta {\tilde{S}}^{\prime }{{\tilde{S}}^{\prime }}^{T}{\tilde{W}}^{-1/2}$ of Equation (43) corresponding to faulty satellites of a fault mode. $\tilde{W}$ is a block diagonal matrix whose *ℓ*th block is $W_{\ell }$, and $\Delta {\underline{\tilde{\rho }}}^{*}$ = ${\tilde{W}}^{-1/2}\Delta \underline{\tilde{\rho }}$. $\Theta_{4}$ is a diagonal matrix with diagonal entries constituted from relevant terms of ${W_{rr,\;\ell }}^{-1}\left(I-G_{\ell }^{rr}\right)$, *ℓ* = *k* (=$p_{1}$), k−1, …, k−M+1 (=$m_{1}$), of Equation (43). $(\mathrm{maximum}\;\;{\mathrm{FMS})}_{\alpha 1,\;i}$ is given by the square root of the maximum eigenvalue of $L\Lambda^{-1}$, $\lambda_{L\Lambda^{-1}}^{\max}$. Since $\Theta_{4}$ is a diagonal matrix, $\Lambda^{-1}$ requires the inverse of an M×M matrix for SF cases and 2M×2M matrix for DF cases. Thus, the upper bound of mean VPE corresponding to $\alpha_{1}$ for fault mode *i* is

(48) $${\mathrm{VPE}}_{i,\;1}^{U}=\sqrt{\lambda_{L\Lambda^{-1}}^{\max}\Gamma }$$

where Γ = ${\underline{f}}_{w}^{T}\Lambda {\underline{f}}_{w}$. It is determined next corresponding to the allocated probability of missed detection for fault mode *i*, $P_{MD,\;i}^{alloc}$. $P_{MD,\;i}^{alloc}$ for SF and DF modes is

(49) $$\begin{array}{cc} P_{MD,\;i}^{alloc} & =P_{HMI,\;3}^{alloc,\;sf}/\left(n^{max}P_{F}\right)\;\left(\mathrm{for}\;\mathrm{SF}\;\mathrm{modes}\right)\\ P_{MD,\;i}^{alloc} & =P_{HMI,\;3}^{alloc,\;df}/\left(0.5n^{max}\left(n^{max}-1\right)P_{F}^{2}\right)\;\left(\mathrm{for}\;\mathrm{DF}\;\mathrm{modes}\right) \end{array}$$

where $n^{max}$ is the maximum number of visible satellites.

Using Equation (35), and putting pseudorange rate measurement error, ${\underline{\eta }}_{4}$ in place of pseudorange rate innovations, $\Delta {\underline{\dot{\rho }}}_{\ell }$ for the same reason as that discussed before Equation (33), Equation (43) for $\alpha_{1}$ is written as

(50) $$\begin{array}{cc} \alpha_{1}^{2} & ={{\underline{\eta }}_{3}}^{T}\Theta_{2}{\underline{\eta }}_{3}+\sum_{\ell =p_{1}}^{m_{1}}\left({\underline{\eta }}_{4,\;\ell }^{T}{W_{rr,\;\ell }}^{-1}\left(I-G^{rr,\;\ell }\right){\underline{\eta }}_{4,\;\ell }\right) \end{array}$$

Replacing $W_{rr,\;\ell }^{-1/2}{\underline{\eta }}_{4,\;\ell }$ with ${\underline{\eta }}_{5,\;\ell }$, and $W_{\mathrm{rr},\;\ell }^{-1/2}H_{\ell }{\left(H_{\ell }^{T}W_{\mathrm{rr},\;\ell }^{-1}H_{\ell }\right)}^{-1}H_{\ell }^{T}W_{\mathrm{rr},\;\ell }^{-1/2}$ with $G_{\ell }^{rr,\;*}$

(51) $$\begin{array}{cc} \alpha_{1}^{2} & ={{\underline{\eta }}_{3}}^{T}\Theta_{2}{\underline{\eta }}_{3}+\sum_{\ell =p_{1}}^{m_{1}}\left({\underline{\eta }}_{5,\;\ell }^{T}\left(I-G_{\ell }^{rr,\;*}\right){\underline{\eta }}_{5,\;\ell }\right) \end{array}$$

Similar to Equation (15)

(52) $$\left(I-G_{\ell }^{rr,\;*}\right)=S_{\ell }^{rr}\left(1:n,1:n-n_{\mathrm{null}}\right){\left(S_{\ell }^{rr}\left(1:n,1:n-n_{\mathrm{null}}\right)\right)}^{T}$$

Replacing $S_{\ell }^{rr}\left(1:n,1:n-n_{\mathrm{null}}\right)$ with ${S_{\ell }^{rr,}}^{\prime }$, and substituting Equation (52) into Equation (51)

(53) $$\alpha_{1}^{2}={{\underline{\eta }}_{3}}^{T}\Theta_{2}{\underline{\eta }}_{3}+\sum_{\ell =p_{1}}^{m_{1}}\left({\underline{\eta }}_{5,\;\ell }^{T}{S_{\ell }^{rr,}}^{\prime }{\left({S_{\ell }^{rr,}}^{\prime }\right)}^{T}{\underline{\eta }}_{5,\;\ell }\right)$$

Putting ${\left(\Theta_{2}\right)}^{1/2}{\underline{\eta }}_{3}$ as ${\underline{\eta }}_{6}$(${\left(\Theta_{2}\right)}^{1/2}$ is the matrix square root of $\Theta_{2}$), and stacking ${\left({S_{\ell }^{rr,}}^{\prime }\right)}^{T}{\underline{\eta }}_{5,\;\ell }$ for all relevant values of *ℓ* in ${\underline{\eta }}_{7}$

(54) $$\alpha_{1}^{2}={{\underline{\eta }}_{6}}^{T}{\underline{\eta }}_{6}+{{\underline{\eta }}_{7}}^{T}{\underline{\eta }}_{7}$$

With known error covariance of pseudorange rate measurements, ${\underline{\eta }}_{7}$ has unit-variance independent elements. Hence, its covariance is identity matrix. Since ${\underline{\eta }}_{3}$ also consists of unit-variance independent elements (see before Equation (34)), the covariance of ${\underline{\eta }}_{6}$ is $\Theta_{2}$. It was shown in the previous subsection that the eigenvalues of $\Theta_{2}$ lie between zero and one. The error covariance of ${\left[{{\underline{\eta }}_{6}}^{T}\;{\underline{\eta }}_{7}^{T}\right]}^{T}$ (=$\underline{\tilde{\eta }}$) denoted as $\tilde{\Theta }$ is a block diagonal matrix. The first block is $\Theta_{2}$, and the second block is identity matrix. Thus, the maximum eigenvalue of $\tilde{\Theta }$, $\lambda_{\tilde{\Theta }}^{\max}$ is unity. The minimum eigenvalue $\lambda_{\tilde{\Theta }}^{\min}$ is greater than zero and equal to the minimum eigenvalue of $\Theta_{2}$. Although not considered in this paper, unknown error covariance of pseudorange rate measurements can be accommodated following [49]. If the mean of $\underline{\tilde{\eta }}$ due to faults is represented as $\underline{\tilde{f}}$, then the probability of missed detection $P_{MD}$ is given by

(55) $$P_{MD}=\int_{{\underline{\tilde{\eta }}}^{T}\underline{\tilde{\eta }}⩽T_{th,\;1}^{2}}N\left(\underline{\tilde{\eta }},\;\tilde{\Theta },\;\underline{\tilde{f}}\right)dV$$

where N stands for multi-variate Gaussian distribution, and *V* is the variable of integration. The square of magnitude of $\underline{\tilde{f}}$, ${\underline{\tilde{f}}}^{T}\underline{\tilde{f}}$, is Γ, which is the same as ${\underline{f}}_{w}^{T}\Lambda {\underline{f}}_{w}$ under fault mode *i* (see after Equation (48)). Let the non-central Chi square distribution $P_{ncx}\left({\left(T_{th,\;1}^{*}\right)}^{2},\mathrm{DOF},\Gamma^{*}\right)$ with non-centrality parameter $\Gamma^{*}$ and for some threshold ${\left(T_{th,\;1}^{*}\right)}^{2}$ have a probability $P_{1}$, then $P_{MD}$ ⩽ $P_{1}$ if [49]

(56) $$\begin{array}{cc} {\left(T_{th,\;1}^{*}\right)}^{2} & =\frac{1}{{\left(\lambda_{\tilde{\Theta }}^{\min}\right)}^{2}}T_{th,\;1}^{2}\\ \sqrt{\Gamma^{*}} & =\left(\sqrt{\Gamma }-T_{th,\;1}\right)+T_{th,\;1}^{*} \end{array}$$

If $\Gamma^{*}$ is obtained such that $P_{ncx}\left({\left(T_{th,\;1}^{*}\right)}^{2},\mathrm{DOF},\Gamma^{*}\right)$ = $P_{MD,\;i}^{alloc}$, DOF = number of rows/columns of $\tilde{\Theta }$, and Γ is calculated from Equation (56), then $P_{MD}$ ⩽ $P_{MD,\;i}^{alloc}$. This value of Γ is used in Equation (48). A look up table of Γ is prepared for different values of $1/{\left(\lambda_{\tilde{\Theta }}^{\min}\right)}^{2}$ and DOF using $P_{MD,\;i}^{alloc}$. It is justified in the beginning of Appendix D that Γ increases with $1/{\left(\lambda_{\tilde{\Theta }}^{\min}\right)}^{2}$. Hence, Γ pertaining to the entry in the look up table next higher than the calculated $1/{\left(\lambda_{\tilde{\Theta }}^{\min}\right)}^{2}$ is considered. The bound on mean VPE for $\alpha_{2}$ under fault mode *i*, $VPE_{2,\;i}^{U}$ is discussed next.

#### 5.2.2. $VPE_{2,\;i}^{U}$

$VPE_{2,\;i}^{U}$ is calculated in a similar way to that of $VPE_{1,\;i}^{U}$. The major differences are as follows. First, it involves terms of (N−M) epochs from $t_{k-M}$ to $t_{k-N+1}$. (N−M) is generally found to be higher than *M*. Therefore, for the calculation of $\Theta_{3}$ of Equation (47), relevant rows of ${\tilde{W}}^{-1/2}{\tilde{S}}_{k}^{\prime }{\tilde{S}}_{k}^{\prime T}$ are identified corresponding to the fault mode and then multiplied with Θ. This way, large matrix multiplications, which take longer time, are avoided. $\Lambda^{-1}$ requires inverting an (N−M)×(N−M) matrix for an SF mode and a 2(N−M)×2(N−M) matrix for a DF mode. In simulation studies with realistic error models for airborne users, this is not found to be a time intensive operation. This will be evident later with algorithm execution times.

Second, Θ is pre-calculated using $\Psi_{\max}$ instead of the actual Ψ to avoid forming a large Ψ over N−M epochs. $\lambda_{\tilde{\Theta }}^{\min}$ of Equation (56) is also calculated with $\Psi_{\max}$ in the place of Ψ. In order to ensure that the corresponding Γ is overbounded, the following is justified in Appendix D. For a given probability $P_{1}$, Γ increases with decreasing $\lambda_{\tilde{\Theta }}^{\min}$, and the calculated $\lambda_{\tilde{\Theta }}^{\min}$ is lower than the actual value obtained with Ψ.

The third bound $VPE_{3}^{U}$ accounts for the contributions of all discarded terms before epoch $t_{k-N+1}$ to the mean position error. Since it remains the same for all fault modes, the fault mode index *i* is dropped from its subscript. It is presented next.

#### 5.2.3. $VPE_{3}^{U}$

As noted earlier, the third test with discarded terms has been eliminated in the current work. However, discarded terms may have an effect on current mean position error at $t_{k}$, which should be bounded by $VPE_{3}^{U}$. In this context, a pertinent question arises as to which faults should be considered for the bound. Suppose at a time epoch $t_{\ell }$, ${\underline{f}}_{w}^{T}\Lambda {\underline{f}}_{w}$ corresponding to a fault detection test is equal to Γ, which is determined from $P_{MD,\;i}^{alloc}$ under fault mode *i*. Hence, for any fault $\underline{f}$ under the same fault mode such that ${\underline{f}}^{T}\Lambda \underline{f}>\Gamma$, the probability of missed detection is less than $P_{MD,\;i}^{alloc}$ at $t_{\ell }$. Positinon error due to this fault is not bounded at $t_{\ell }$. It also need not be bounded at subsequent epochs. This is because if HMI occurs at $t_{\ell }$ due to this fault with missed detection probability less than $P_{MD,\;i}^{alloc}$, then bounding its contribution to position error at subsequent epochs holds little meaning. It can be explained with the help of the relative frequency approach of the probability theory. Thus, $VPE_{3}^{U}$ at $t_{k}$ bounds the contributions of faults with probability of missed detection more than the allocated probability corresponding to both $\alpha_{1}$ and $\alpha_{2}$ and all fault modes over all epochs from $t_{1}$ up to $t_{k-N}$. It should be noted that $VPE_{2,\;i}^{U}$, if obtained in a way similar to that of $VPE_{3}^{U}$, would be too large and inflate the PL to a great extent. Therefore, $VPE_{2,\;i}^{U}$ is not determined this way. ${\mathrm{VPE}}_{3}^{U}$ is [33]

(57) $${\mathrm{VPE}}_{3}^{U}=U_{\max}N_{\max}\times \frac{1\times 10^{-6}}{1-0.1}\left({\hat{b}}_{\max}+{\dot{\hat{b}}}_{\max}\right)$$

For the HPE, the factor $1\times 10^{-6}$ is modified to $\sqrt{2}\times 10^{-6}$ to include north and east components. $N_{\max}$ is the maximum of all values of *N* up to $t_{k}$. *N* at an epoch is determined in the following way for an asymptotically stable filter. At an epoch $t_{k}$, the last B matrix (see after Equation (44) for definition) with 2-norm larger than or equal to $1\times 10^{-6}$ is identified. If the magnitude of *N* at $t_{k-1}$ is more than the index of that B matrix, *N* is set to that index plus one. One is added to account for the new term at $t_{k}$. All B matrices with indices higher than updated (N−1) are not considered anymore. On the other hand, if the index is the same as *N* at $t_{k-1}$, *N* is only increased by one to include the new term at $t_{k}$. All terms including B matrices from epochs before $t_{k-N+1}$ are discarded or deleted from memory. Note that a small threshold $1\times 10^{-6}$ is chosen to calculate *N*. A smaller value can also be chosen, but that will increase *N*. $U_{\max}$ is an upper bound such that a discarded B matrix cannot have a 2-norm outside the range of $U_{\max}\times 10^{-6}$ and zero after it is discarded. A computationally efficient algorithm to find $U_{\max}$ at each epoch is provided in [33]. The denominator of Equation (57) results from an infinite geometric series with common ratio 0.1. Next, the maximum values of bias and bias rate, ${\hat{b}}_{\max}$ and ${\dot{\hat{b}}}_{\max}$, respectively from faults mentioned before Equation (57) are calculated differently in this paper as follows.

(58) $$\begin{array}{cc} {\hat{b}}_{\max} & =\sqrt{\Gamma_{\max}/s_{\min}},\;{\dot{\hat{b}}}_{\max}=2\sqrt{\Gamma_{\max}/s_{\min}^{\prime }} \end{array}$$

Each term of the preceding equation is obtained next. $\Gamma_{\max}$ is the maximum of all Γs calculated for SF and DF modes corresponding to $\alpha_{1}$ and $\alpha_{2}$ over all epochs up to $t_{k}$. Using ${\underline{f}}_{w}^{T}\Lambda {\underline{f}}_{w}$ = Γ, and the fact that $\Theta_{4}$ of Λ in Equation (47) is a diagonal matrix, $s_{\min}^{\prime }$ is

(59) $$s_{\min}^{\prime }=\min_{\forall \;\ell =1,\cdots ,k}\min_{\alpha_{1},\;\alpha_{2}}\min_{i\in \mathrm{fault}\;\mathrm{modes}}(\min\left(\mathrm{diag}\left(\Theta_{4}\right)\right)$$

where the operator “min” denotes minimum over all terms provided below it or within parentheses next to it, and diag(.) has all diagonal elements of a square matrix. It should be noted that the multiplication of two in ${\dot{\hat{b}}}_{\max}$ of Equation (58) accounts for DF modes (i.e., faults in any two pseudorange rate measurements).

In order to determine ${\hat{b}}_{\max}$, $\Theta_{3}$ of Λ in Equation (47) is used. If ${\underline{f}}_{\rho }$ represents faults in only pseudorange measurements for a fault mode, then one can write

(60) $$\begin{array}{cc} {\underline{f}}_{\rho }^{T}\Theta_{3}{\underline{f}}_{\rho }⩽ & \;\Gamma_{\max} \end{array}$$

Multiplying and dividing ${\underline{f}}_{\rho }^{T}{\underline{f}}_{\rho }$ on the left-hand side

(61) $$\begin{array}{cc} \frac{{\underline{f}}_{\rho }^{T}\Theta_{3}{\underline{f}}_{\rho }}{{\underline{f}}_{\rho }^{T}{\underline{f}}_{\rho }}\left({\underline{f}}_{\rho }^{T}{\underline{f}}_{\rho }\right)⩽ & \;\Gamma_{\max} \end{array}$$

Noting that the minimum value of $\frac{{\underline{f}}_{\rho }^{T}\Theta_{3}{\underline{f}}_{\rho }}{{\underline{f}}_{\rho }^{T}{\underline{f}}_{\rho }}$ is the minimum eigenvalue of $\Theta_{3}$, $\lambda_{\Theta ,\;3}^{\min}$, ${\hat{b}}_{\max}^{2}$(or $\max\left({\underline{f}}_{\rho }^{T}{\underline{f}}_{\rho }\right)$) is given by

(62) $$\begin{array}{cc} \left(\min_{\forall \;\ell =1,\cdots ,k}\min_{\alpha_{1},\;\alpha_{2}}\min_{i\in \mathrm{fault}\;\mathrm{modes}}\lambda_{\Theta ,\;3}^{\min}\right){\hat{b}}_{\max}^{2} & =\Gamma_{\max} \end{array}$$

It is apparent that for ${\hat{b}}_{\max}$, the two need not be multiplied as $\left({\underline{f}}_{\rho }^{T}{\underline{f}}_{\rho }\right)$ includes DF. Next, the PLs are determined.

### 5.3. Protection Levels (PLs)

The VPL is determined in this subsection without assuming statistical independence between test statistics and position error. The HPL can also be calculated the same way. The probabilities of HMI for the vertical position under NF, SF and DF are $P_{HMI,\;3}^{nf}$, $P_{HMI,\;3}^{sf}$ and $P_{HMI,\;3}^{df}$, respectively. Their allocated values are provided in Section 3. If $\epsilon_{v}$ represents the absolute VPE, and ${\mathrm{VPL}}_{NF}$ is the VPL under NF, then by definition, $P_{HMI,\;3}^{nf}$ is

(63) $$\begin{array}{cc} P_{HMI,\;3}^{nf} & =P(\left(\epsilon_{v}>{\mathrm{VPL}}_{NF}\right)\cap \left(\left(\alpha_{1}⩽T_{th,\;1}\right)\cap \left(\alpha_{2}⩽T_{th,\;2}\right)\right)|NF)\\ \; & =P\left(\left(\epsilon_{v}>{\mathrm{VPL}}_{NF}\right)|NF\right)P((\left(\alpha_{1}⩽T_{th,\;1}\right)\cap \\ & \left(\alpha_{2}⩽T_{th,\;2}\right))|\left(\epsilon >{\mathrm{VPL}}_{NF}\right)\left|NF\right)\\ & ⩽P(\left(\epsilon_{v}>{\mathrm{VPL}}_{NF}\right)|NF) \end{array}$$

Thus, ${\mathrm{VPL}}_{NF}$ is obtained by setting $P(\left(\epsilon_{v}>{\mathrm{VPL}}_{NF}\right)|NF)$ equal to $P_{HMI,\;3}^{alloc,\;nf}$.

For an SF mode, $P_{HMI,\;3}^{sf}$ is given by

(64) $$\begin{array}{cc} P_{HMI,\;3}^{sf} & =P\left(\left(\epsilon_{v}>{\mathrm{VPL}}_{SF}\right)\cap \left(\left(\alpha_{1}⩽T_{th,\;1}\right)\cap \left(\alpha_{2}⩽T_{th,\;2}\right)\right)|SF\right)n_{f}^{s}P_{F} \end{array}$$

where $n_{f}^{s}$ is the number of SF modes, VPL${}_{SF}$ is the VPL under SF, and $P_{F}$ is the prior probability of a satellite fault defined in Section 3. Representing the probability of missed detection for all SF modes as $P_{MD,\;sf}$ and following the same approach as before

(65) $$\begin{array}{cc} P_{MD,\;sf} & =\frac{P_{HMI,\;3}^{sf}}{n_{f}^{s}P_{F}}=P\left(\left(\epsilon_{v}>{\mathrm{VPL}}_{SF}\right)\cap \left(\left(\alpha_{1}⩽T_{th,\;1}\right)\cap \left(\alpha_{2}⩽T_{th,\;2}\right)\right)|SF\right)\\ \mathrm{or}, & \;P_{MD,\;sf}⩽P\left(\alpha_{1}⩽T_{th,\;1}|SF\right)\\ \mathrm{or}, & \;P_{MD,\;sf}⩽P\left(\alpha_{2}⩽T_{th,\;2}|SF\right) \end{array}$$

Γs for $\alpha_{1}$ and $\alpha_{2}$ are determined such that $P(\alpha_{1}⩽T_{th,\;1}|SF)$≤$P_{MD,\;sf}^{alloc}$, and $P(\alpha_{2}⩽T_{th,\;2}|SF)$≤$P_{MD,\;sf}^{alloc}$, respectively (see after Equation (56)). $P_{MD,\;sf}^{alloc}$ is equal to $\frac{P_{HMI,\;3}^{alloc,\;sf}}{n_{f}^{s,\;\max}P_{F}}$. $n_{f}^{s,\;\max}$ is the maximum value of $n_{f}^{s}$. VPL${}_{SF}$ is

(66) $${\mathrm{VPL}}_{SF}=\sum_{j=1}^{2}\left(\max_{i\in \mathrm{SF}\;\mathrm{modes}}VPE_{j,\;i}^{U}\right)+VPE_{3}^{U}+\gamma_{s}\sigma_{v}$$

where the operator “max” denotes maximum over all terms provided below it or within parentheses next to it. $P(\epsilon_{v}>\gamma_{s}\sigma_{v})$ = $P_{MD,\;sf}^{alloc}$. $\sigma_{v}$ is the error standard deviation of the estimated vertical position.

Similarly for DF modes, the probability of missed detection, $P_{MD,\;df}$ is

(67) $$\begin{array}{cc} P_{MD,\;df} & ⩽P(\alpha_{1}⩽T_{th,\;1}|DF)\\ \mathrm{or},\;P_{MD,\;df} & ⩽P(\alpha_{2}⩽T_{th,\;2}|DF) \end{array}$$

The corresponding Γs are also calculated for DF modes. The VPL for DF modes, VPL${}_{DF}$ is

(68) $${\mathrm{VPL}}_{DF}=\sum_{j=1}^{2}\left(\max_{i\in \mathrm{DF}\;\mathrm{modes}}VPE_{j,\;i}^{U}\right)+VPE_{3}^{U}+\gamma_{d}\sigma_{v}$$

where $P(\epsilon_{v}>\gamma_{d}\sigma_{v})$ = $P_{MD,\;df}^{alloc}$. $P_{MD,\;df}^{alloc}$ = $\frac{P_{HMI,\;3}^{alloc,\;df}}{n_{f}^{d,\;\max}P_{F}}$, where $n_{f}^{d}$ is the number of DF modes, and $n_{f}^{d,\;\max}$ is its maximum value.

The final VPL is

(69) $$\mathrm{VPL}=\max({\mathrm{VPL}}_{NF},\;{\mathrm{VPL}}_{SF},\;{\mathrm{VPL}}_{DF})$$

## 6. Simulation Studies

Primary coverage area of NavIC over the Indian sub-continent is chosen for simulation studies in this paper. A UAV is assumed to fly at an altitude of 184 m above the WGS84 reference ellipsoid at speed 10 m/s with a constant heading of −45${}^{\circ }$ for 20 min. UAV speed, altitude and flying time chosen are commensurate with those of small unmanned aircraft [51]. The starting point of the flight path is varied from 0${}^{\circ }$ to 40${}^{\circ }$ N latitudes and 60${}^{\circ }$ E to 105${}^{\circ }$ E longitudes in steps of 1${}^{\circ }$. There are a total of 1886 simulations, each of duration 20 min. Every 50 simulations, the start time is increased by 7.5 h. At a time, maximum twelve GPS satellites are simulated due to channel constraints of the simulation platform. Dual frequency GPS (L${}_{1}$ and L${}_{5}$) and NavIC (L${}_{5}$ and S) measurements are generated for the UAV flight path. Error models of carrier smoothed pseudorange measurements are discussed in Section 4 while describing SKF. White noise covariance of pseudorange rate measurements is obtained from the tracking error of a second order frequency locked loop (FLL) for 45 dB-Hz C/N${}_{0}$. The FLL is assumed to have a cross product discriminator, a coherent integration time of 10 ms and two-sided noise equivalent bandwidth of 4 Hz. Power spectral density of a temperature controlled crystal oscillator is considered for simulation of receiver clock error. Measurement update interval of both WLS and KF is one second.

**Figure 5:**
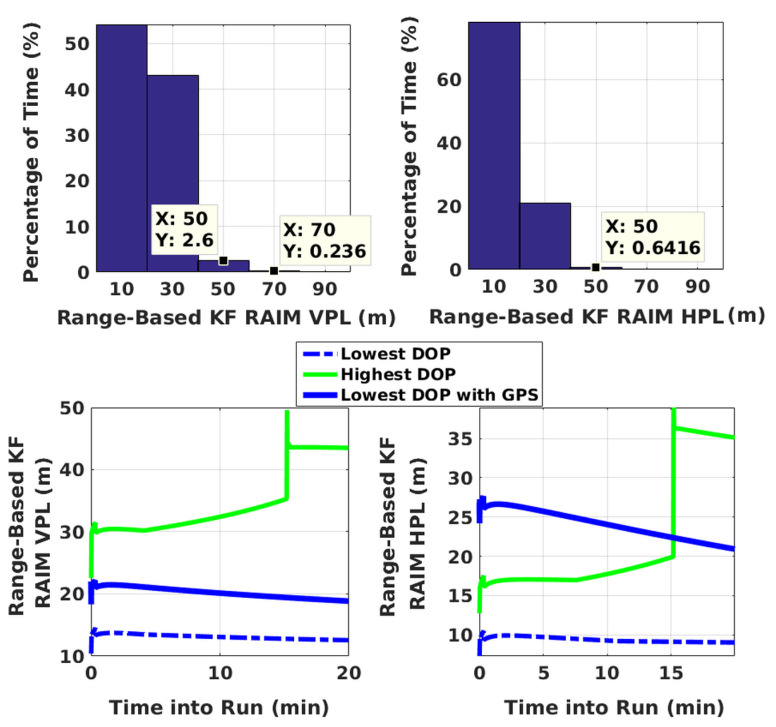
Histogram plots of PLs of KF range-based RAIM, and time evolution of KF PLs for highest and lowest GDOP scenarios. “Lowest DOP with GPS” indicates GPS-only scenario.

VPLs and HPLs of the developed range-based KF RAIM algorithm are illustrated in Figure 5. Both GPS and NavIC constellations are considered. The geometric dilution of precision (GDOP) varies between 0.5 and 3.5. It is within 2.5 for 97.6% of the time as against 73.3% with GPS alone. For illustration purposes, PLs calculated every second are grouped into five bins, whose center points are indicated along the x-axis. Percentage of time along the y-axis is calculated as the ratio of the number of time instants at which the PL falls within a given range to the total number of time instants over all 1886 simulations times one hundred. KF VPL and HPL are within 40 m 97.16% and 99.35% of the time, respectively.

**Figure 6:**
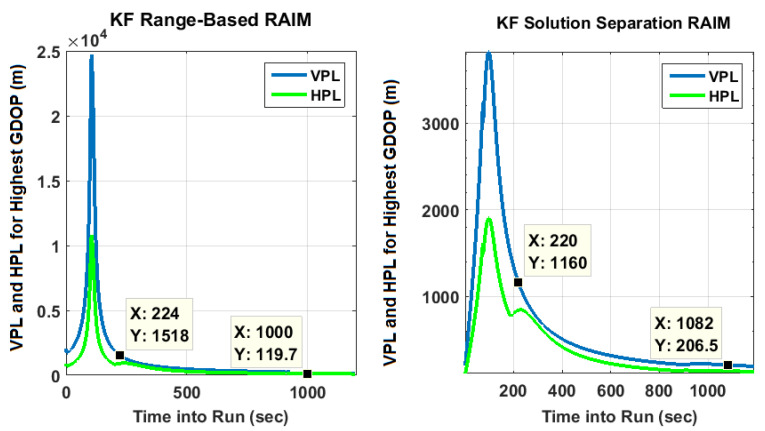
KF PLs with standalone GPS for scenario having the highest GDOP with dual constellations, assuming only single satellite fault.

**Figure 7:**
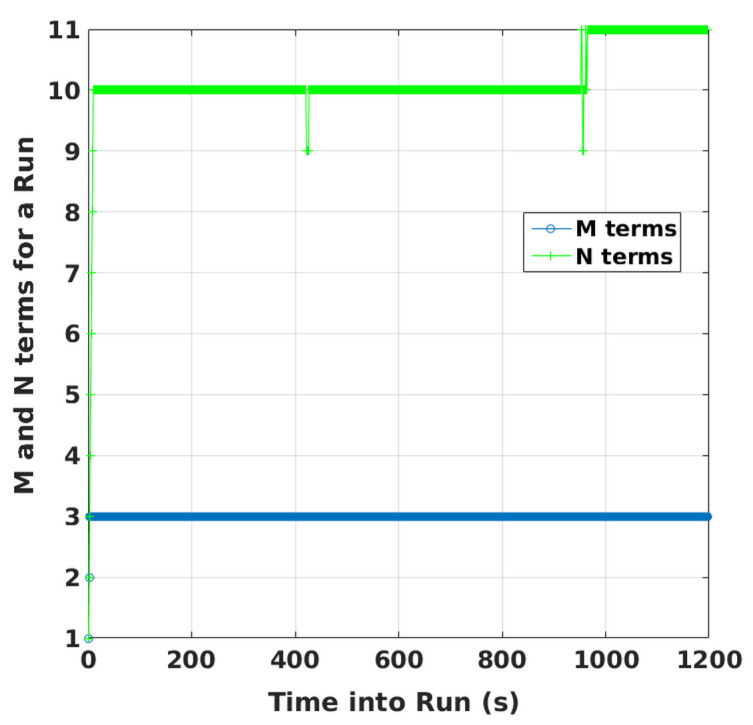
Numbers of *M* and *N* terms of range-based KF RAIM for a simulation.

Evolution of PLs with time are also plotted in Figure 5 for specific GPS plus NavIC and GPS-only cases. For clarity, only two scenarios are selected. They have highest and lowest GDOPs corresponding to dual constellations. With the lowest GDOP, PLs are reduced by about 5 m to 15 m when both constellations are used as compared to PLs for GPS alone. For the highest GDOP, PLs are hundreds of thousands of meters with standalone GPS (having five-six visible satellites). This is shown separately in Figure 6 along with PLs of KF solution separation RAIM, with the assumption of a single satellite fault. Although not shown, a few other high GDOP cases are studied and found to have similar performance with GPS. Thus, it can be concluded that improvements obtained by using NavIC alongside GPS are more significant in poor geometries. *M* and *N* terms of KF range-based RAIM for a simulation are illustrated in Figure 7. They are of the same order in all simulations.

**Figure 8:**
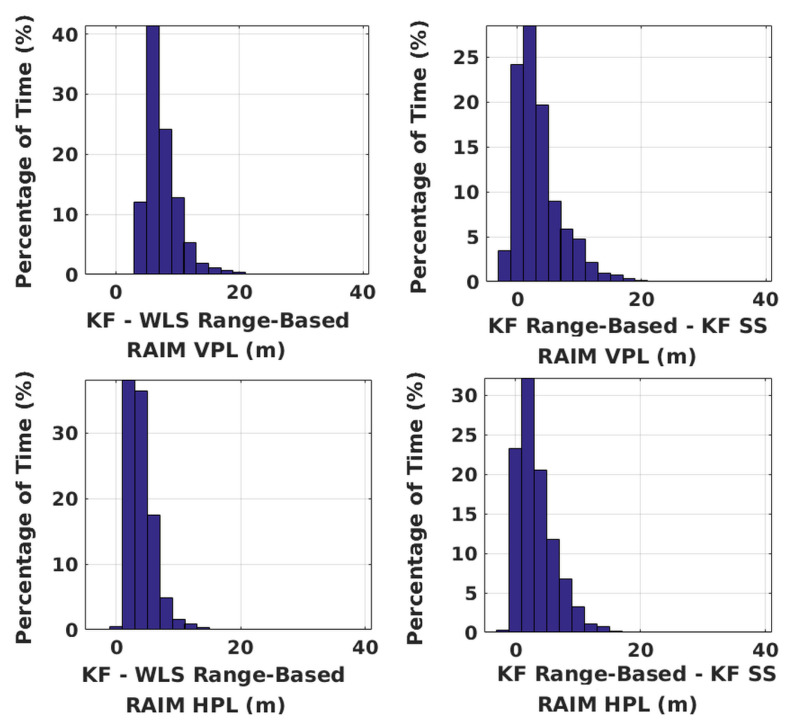
Performance comparison of all integrity algorithms over all simulations with GPS and NavIC. SS stands for solution separation. Along the x-axis, the difference between the center points of any two adjacent bins is 2 m.

Having shown the PLs of range-based KF RAIM, its performance is compared against WLS range-based RAIM and KF solution separation RAIM using both constellations. Figure 8 depicts comparison results. Range-based KF RAIM has VPL within 3 to 11 m of that of range-based WLS RAIM 90.4% of the time and HPL within −1 to 11 m 98.82% of the time. Its VPL, on the other hand, is within −3 to 11 m of that of its solution separation counterpart 95.49% of the time, and HPL is within −3 to 11 m 97.28% of the time. Using a vertical alert limit of 50 m and horizontal alert limit of 40 m, availability of the three algorithms is shown in Table 1. Availability is calculated as the percentage of simulations, in which the PL is below the corresponding alert limit for the whole duration of 20 min. PL depends on both $P_{FA}$ and $P_{HMI}$, for which no proper values have yet been fixed for UAV applications. Thus, availability can only be treated as another way of comparing algorithm performances.

It is apparent from the table that the two existing algorithms offer higher availability than range-based KF RAIM. It is observed that its PL sometimes exhibits an instantaneous change when a satellite sets or rises. If such a change can be smoothed out by some method, its availability would increase to 98.46% in the vertical direction and 98.89% in the horizontal direction. This will be carried out in future work.

All 1886 simulations were run in MATLAB on a DELL core i7-8700 (CPU @ 3.2 GHz) computer with a Linux operating system, and with 16 GB RAM and 20 GB swap memory. WLS range-based RAIM took 11 h to complete all simulations. Range-based KF RAIM algorithm needed 3 days and 16 h whereas KF solution separation RAIM required 9 days. In order to see execution times of the algorithms on a low end computer, time needed by them for a simulation on an IBM Thinkpad laptop is also noted. The laptop has Linux operating system, core 2 duo processor (CPU @ 2 GHz), 1 GB RAM and 2 GB swap memory. The three algorithms needed 20 s, 600 s and 1521 s, respectively, for a 20-min (1200 s) run. Time includes that needed for running the navigation filter as well. A few other simulations also took similar times. MATLAB tic and toc functions are used to record execution times.

Based on the above performance analyses, it can be concluded that WLS has the lowest execution time. However, since WLS is not preferred in advanced navigation methods, as noted earlier, KF-based RAIM algorithms are essential to ensuring reliability of such systems. Of the two KF algorithms, solution separation RAIM provides lower PLs, but has a much higher computational load. The computational burden can be reduced by fault grouping at the expense of inflated PLs [28].

Range-based KF RAIM, on the other hand, offers satisfactory performance to a certain extent with moderate computational resources, thereby having potential for real-time implementations. Its comparatively large PL is partially attributed to the fact that SKF is sub-optimal in nature for not estimating the GM states. SKF formulation is necessary for reasons discussed earlier. It allows the formation of more than one test statistic using innovation terms of a finite number of epochs and a separate FMS for each statistic. This prevents the PL from growing with time, which is considered a major challenge with KF RAIM in the range domain. There may be some scope of fine tuning the algorithm further for lower PLs, which will be attempted in the future.

## 7. Conclusions

A novel range domain KF RAIM algorithm is presented in this paper, building upon previous work of the authors. Major changes in the current algorithm include modeling time correlated errors of the pseudorange measurements and accounting for multi-satellite failures. The integrity monitoring performance of the developed algorithm is studied for a UAV trajectory with GPS and NavIC over the primary coverage area of the latter. It is shown that both WLS range-based and KF solution separation RAIM algorithms outperform the new method. However, WLS RAIM has limitations in that it is not ideal for advanced navigation systems. KF solution separation RAIM, on the other hand, offers lower PLs than the range-based KF algorithm, but with a much higher computational load. PLs of range-based KF RAIM are within 10 m of those of KF solution separation RAIM at least 95% of the time. Due to its low execution times, range-based KF RAIM shows promise for real-time implementations in avionics. It also has potential for extensions to advanced methods such as vector receivers, where running parallel vector loop filters for solution separation RAIM would be prohibitively expensive. Further, simulation results indicate that addition of NavIC alongside GPS can substantially improve RAIM performance, particularly in poor geometries.

Future extensions of the current work include extensive testing of RAIM for various geometries with GPS and NavIC over the latter’s primary and secondary coverage areas for different dynamic scenarios. A suitable error model also needs to be developed for NavIC and validated with real satellite data. The error model will be used for experimental validation of the RAIM algorithms. In addition, it is important to take into account constellation-wide faults for integrity monitoring with multiple constellations. While this has been addressed in the literature for solution separation or ARAIM, a suitable methodology has to be devised for range-based RAIM. It is noted that the developed KF RAIM algorithm at times exhibits an instantaneous change in PL when satellite visibility changes. This should be smoothed out with appropriate modifications. Fine tuning of the algorithm will also be attempted to reduce PLs. Effects of different $P_{FA}$ and $P_{HMI}$ on the proposed method will be investigated. Moreover, the algorithm should be able to deal with unknown error covariances. Its performance in poor signal environments also warrants further studies. Extending the developed KF RAIM to sensor integration and vector tracking is another important area that needs to be explored in depth.

## Figures and Tables

**Table 1:** RAIM availability in percentage with GPS and NavIC.

Position	WLS Range-	KF Range-	KF Solution
	Based RAIM	Based RAIM	Separation
			RAIM
Vertical	99.63	97.4	99.68
Horizontal	99.63	97.4	99.63

**Figure A1:**
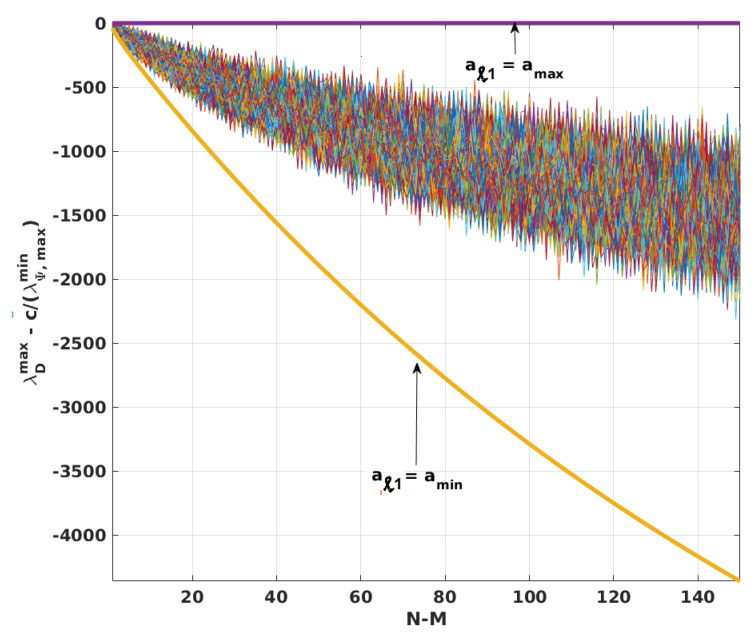
Comparison of $\lambda_{D}^{\max}$ and $\frac{c}{\lambda_{\Psi ,\;\max}^{\min}}$ with different (N−M). Lower and upper limits are shown ∀$a_{\ell 1}$ = $a_{min}$ and $a_{\ell 1}$ = $a_{max}$, respectively. This figure can be regenerated for $\Psi_{\mathrm{sub}}$ with the Matlab program, matlab_prog_figures_3_A1.m provided in the Supplementary Materials.

## Supplementary Materials

The following are available online at https://www.mdpi.com/1424-8220/20/22/6606/s1, matlab_prog_figures_3_A1.m: Matlab program to reproduce Figure 3 and Figure A1; matlab_prog_min_lambda_theta.m: Matlab program to show that the calculated $\lambda_{\tilde{\Theta }}^{\min}$ for $\alpha_{2}$ is less than its actual value. For this, please see the last paragraph of Appendix D.

## Appendix A. Solution Separation KF RAIM

Key equations of solution separation KF RAIM are provided here. The position error bounds, ${\mathrm{PE}}_{SS}^{KF}$ for north, east and down components for NF, SF and DF modes are given by [27]

(A1) $$\begin{array}{cc} {\mathrm{PE}}_{SS,\;h}^{KF,\;0} & =Q^{-1}\left[\frac{P_{HMI,\;h}^{alloc,\;nf}}{2}\right]\sigma_{h}^{0},\;{\mathrm{PE}}_{SS,\;h}^{KF,\;i}=T_{SS,\;h}^{i}+Q^{-1}\left[\frac{P_{HMI,\;h}^{alloc,\;sf}}{2n_{f}^{s}P_{F}}\right]\sigma_{h}^{i}\\ {\mathrm{PE}}_{SS,\;h}^{KF,\;j} & =T_{SS,\;h}^{j}+Q^{-1}\left[\frac{P_{HMI,\;h}^{alloc,\;df}}{2n_{f}^{d}P_{F}^{2}}\right]\sigma_{h}^{j} \end{array}$$

where *h* = 1, 2 and 3, denote north, east and down components, respectively. *Q* is the tail probability of zero mean unit normal distribution given by $\frac{1}{\sqrt{2\pi }}\int_{q}^{\infty }e^{-q^{2}/2}dq$, and $Q^{-1}$ is its inverse. “0” represents NF condition. *i* (or *j*) denotes a fault mode with the corresponding EKF using a subset of all measurements. The subset is determined by excluding one (or two) satellite(s) assumed faulty in the fault mode. $\sigma_{h}^{i}$ (or $\sigma_{h}^{j}$) is the corresponding standard deviation for north, east or down component of position. $\sigma_{h}^{0}$ is the standard deviation for all-in-view case when no satellites are assumed faulty. $n_{f}^{s}$ is the number of single-satellite fault modes. $n_{f}^{d}$ is the number of dual-satellite fault modes. $T_{SS}^{i}$ (or $T_{SS}^{j}$) is the fault detection threshold for fault mode *i* (or *j*). The HPL and VPL are given by

(A2) $$\begin{array}{cc} {\mathrm{HPL}}_{SS}^{KF} & =\max[\sqrt{{\left({\mathrm{PE}}_{SS,\;1}^{KF,\;0}\right)}^{2}+{\left({\mathrm{PE}}_{SS,\;2}^{KF,\;0}\right)}^{2}},\\ & \max_{i}\left[\sqrt{({\mathrm{PE}}_{SS,\;1}^{KF,\;i}{)}^{2}+({\mathrm{PE}}_{SS,\;2}^{KF,\;i}{)}^{2}}\right],\\ \; & \max_{j}\left[\sqrt{{\left({\mathrm{PE}}_{SS,\;1}^{KF,\;j}\right)}^{2}+{\left({\mathrm{PE}}_{SS,\;2}^{KF,\;j}\right)}^{2}}\right]] \end{array}$$

(A3) $$\begin{array}{cc} {\mathrm{VPL}}_{SS}^{KF} & =\max\left[{\mathrm{PE}}_{SS,\;3}^{KF,\;0},\;\max_{i}\left[{\mathrm{PE}}_{SS,\;3}^{KF,\;i}\right],\max_{j}\left[{\mathrm{PE}}_{SS,\;3}^{KF,\;j}\right]\right] \end{array}$$

The threshold for a fault mode *i* (SF) or *j* (DF) is

(A4) $$T_{SS,\;h}^{i/j}=Q^{-1}\left[\frac{P_{FA}}{3\times 2\times N_{fault}}\right]\sqrt{\left({\left(\sigma_{h}^{i/j}\right)}^{2}-{\left(\sigma_{h}^{0}\right)}^{2}\right)}$$

where $N_{fault}$ is the total number of fault modes. PLs are calculated when test statistic $|{\hat{\underline{\varkappa }}}_{n}^{i/j}\left(h\right)-{\hat{\underline{\varkappa }}}_{n}^{0}\left(h\right)|$ is less than respective threshold $T_{SS,\;h}^{i/j}$∀*i*, *j*. |.| represents the absolute value. ${\hat{\underline{\varkappa }}}_{n}^{i/j}\left(h\right)$ is the *i*th or *j*th subset filter estimate, with *h* = 1, 2 and 3 for north, east and down components of position, respectively. ${\hat{\underline{\varkappa }}}_{n}^{0}\left(h\right)$ denotes the corresponding all-in-view estimate. In this context, it should be noted that a change is made to Equation (A4). It is found that the $\left({\left(\sigma_{h}^{i/j}\right)}^{2}-{\left(\sigma_{h}^{0}\right)}^{2}\right)$ term of the equation results in the test statistics crossing their respective thresholds a few times even in no fault scenarios, which is not statistically consistent. Therefore, it is changed to $\left({\left(\sigma_{h}^{i/j}\right)}^{2}+{\left(\sigma_{h}^{0}\right)}^{2}\right)$ to overbound the distribution.

## Appendix B. SKF Process and Measurement Models and Key Equations

The process and linearized measurement models of ${\underline{X}}_{k}$ (=${\left[\Delta {\underline{\varkappa }}_{k-1}^{T}\;{\underline{p}}_{gm,\;k-1}^{T}\right]}^{T}$) for SKF are

(A5) $$\begin{array}{cc} {\underline{X}}_{k} & =\left[\begin{array}{cc} F_{s} & {\left[0\right]}_{10\times n}\\ {\left[0\right]}_{n\times 10} & F_{p} \end{array}\right]{\underline{X}}_{k-1}+{\underline{w}}_{k-1}\\ \left[\begin{array}{c} \Delta {\underline{\rho }}_{k}\\ \Delta {\dot{\underline{\rho }}}_{k} \end{array}\right] & =C_{k}{\underline{X}}_{k}+{\underline{\xi }}_{k}=\left[\begin{array}{cc} C_{s,\;k} & C_{p,\;k} \end{array}\right]{\underline{X}}_{k}+{\underline{\xi }}_{k} \end{array}$$

where the subscript “s” is for estimated states. Subscript “p” represents GM states, which are not estimated in SKF and treated as “consider” parameters. $F_{s}$ is the state transition matrix of $\Delta \underline{\varkappa }$ and linear for position and velocity vectors expressed in ECEF [52]. $F_{p}=\exp^{\left(-\beta \Delta t\right)}I_{n\times n}$, Δt = measurement update interval = 1 s. β is the inverse of time constant of the GM process. It is 100 s (smoothing interval of carrier smoothed pseudorange measurements). ${\underline{w}}_{k-1}$ denotes process noise vector at $t_{k-1}$, which is distributed as Gaussian with zero mean and covariance Q. ${\underline{\xi }}_{k}$ is the measurement noise vector assumed to be Gaussian distributed with zero mean and covariance $W^{\prime \prime }$. Q and $W^{\prime \prime }$ are given by

(A6) $$\begin{array}{cc} Q & =\left[\begin{array}{cc} Q_{s} & {\left[0\right]}_{10\times n}\\ {\left[0\right]}_{n\times 10} & Q_{p} \end{array}\right],W^{\prime \prime }=\left[\begin{array}{cc} 10^{-6}I_{n\times n} & {\left[0\right]}_{n\times n}\\ {\left[0\right]}_{n\times n} & W_{rr} \end{array}\right] \end{array}$$

$Q_{s}$ is determined based on prior information about user dynamics and receiver clock power spectral density. $Q_{p}$ is a diagonal matrix. Its *i*th diagonal term is given by $\left(1-{\left(\exp^{\left(-\beta \Delta t\right)}\right)}^{2}\right)\times \left({\left(\sigma_{\mathrm{ura}}^{i}\right)}^{2}+{\left(\sigma_{\mathrm{tr}}^{i}\right)}^{2}+{\left(\sigma_{\mathrm{mp}}^{i}\right)}^{2}+{\left(\sigma_{n}^{i}\right)}^{2}\right)$ so that the standard deviation of the *i*th GM state is equal to $\sqrt{{\left(\sigma_{\mathrm{ura}}^{i}\right)}^{2}+{\left(\sigma_{\mathrm{tr}}^{i}\right)}^{2}+{\left(\sigma_{\mathrm{mp}}^{i}\right)}^{2}+{\left(\sigma_{n}^{i}\right)}^{2}}$ at steady state. In $W^{\prime \prime }$, a small variance of $10^{-6}$ is used for pseudorange measurements to avoid numerical issues with the SKF implementation. When a satellite sets or a new satellite comes into visibility, the variance is increased to 0.05 for 10 s to deal with transients. $W_{rr}$ is the pseudorange rate error covariance matrix whose diagonal terms are appropriately chosen for a C/N${}_{0}$ of 45 dB-Hz. The measurement model matrix $C_{s,\;k}$ is defined in [52]. It has additional columns for receiver clock bias and drift of NavIC, in addition to those for GPS. $C_{p,\;k}$ = $\left[\begin{array}{c} I_{n\times n}\\ {\left[0\right]}_{n\times n} \end{array}\right]$. Its dimension is provided in the subscript. The a priori error covariance matrix $\Sigma^{-}$ and Kalman gain K can be partitioned for estimated states and “consider” parameters as

(A7) $$\begin{array}{cc} \Sigma_{k}^{-} & =\left[\begin{array}{cc} \Sigma_{ss,\;k}^{-} & \Sigma_{sp,\;k}^{-}\\ \Sigma_{ps,\;k}^{-} & \Sigma_{pp,\;k}^{-} \end{array}\right],K_{k}=\left[\begin{array}{c} K_{s,\;k}\\ {\left[0\right]}_{n\times 2n} \end{array}\right] \end{array}$$

it should be noted that zero rows of the Kalman gain matrix correspond to GM states ${\underline{p}}_{gm}$. The a posteriori error covariance is given by

(A8) $$\Sigma_{k}^{+}=\left[\begin{array}{cc} \Sigma_{ss,\;k}^{-}-K_{s,\;k}R_{k}K_{s,\;k}^{T} & \Sigma_{sp,\;k}^{-}-K_{s,\;k}C_{k}\left[\begin{array}{c} \Sigma_{sp,\;k}^{-}\\ \Sigma_{pp,\;k}^{-} \end{array}\right]\\ \Sigma_{ps,\;k}^{-}-{\left[\begin{array}{c} \Sigma_{sp,\;k}^{-}\\ \Sigma_{pp,\;k}^{-} \end{array}\right]}^{T}C_{k}^{T}K_{s,\;k}^{T} & \Sigma_{pp,\;k}^{-} \end{array}\right]$$

$R_{k}$ is given by $\left(C_{k}\Sigma_{\;k}^{-}C^{T}\;+\;W^{\prime \prime }\right)$. A numerically stable forward recursive modified rank-one update algorithm to find $\Sigma_{\;k}^{+}$ is implemented in this paper, following [43]. Its derivation can be found in [48]. $K_{s,\;k}$ is derived from $\Sigma_{\;k}^{-}$, and also recursively calculated. Time update steps are the same as those of an EKF. The measurement update equation of SKF is given by

(A9) $$\begin{array}{cc} {\underline{\varkappa }}_{k}^{+} & =\;{\underline{\varkappa }}_{k}^{-}+K_{s,\;k}\left[\left[\begin{array}{c} \Delta {\underline{\rho }}_{k}\\ \Delta {\dot{\underline{\rho }}}_{k} \end{array}\right]-\left(C_{s,\;k}{\underline{\varkappa }}_{k}^{-}+C_{p,\;k}{\underline{p}}_{gm,\;k}^{-}\right)\right] \end{array}$$

(A10) $$\begin{array}{cc} {\underline{p}}_{gm,\;k}^{+} & =\;{\underline{p}}_{gm,\;k}^{-} \end{array}$$

Since GM states are initialized to zero, they always remain zero in the SKF implementation.

## Appendix C. Algorithm to Find Θ for α 1

The algorithm to find Θ is described next.

**Algorithm A1:** Determination of Θ for $\alpha_{1}$
% Add/remove previous epoch terms as needed in $\Theta_{k-1}$ so that it consists of terms of % (M−1) epochs excluding current epoch *k*. This accounts for change in *M* at every epoch. % Reference [50] details a computationally efficient algorithm of how to update the inverse of % a matrix, if a number of rows and columns of the matrix are removed. This is used when *M* % reduces at *k*. For addition of terms when *M* increases, part of the steps described next is % used to update $\Theta_{k-1}$ after minor modifications. Based on the updated $\Theta_{k-1}$, $\Theta_{k}$ is found as % follows. % Form Ψ(1:n,1:end) (i.e., rows of Ψ for the current epoch) index = 1 % Set a flag for condition check *c* = 0.05 % Set initial *c* as 0.05 factor = 9 % It is used to find *c* $\lambda_{\Omega }^{\max}$ = 1.5 % Set $\lambda_{\Omega }^{\max}$ to a value greater than one while ($\lambda_{\Omega }^{\max}$ > 1) if (index == 1) % Find $\Theta_{k}$ by adding current epoch terms; $\Theta_{k}$ is partitioned as ${\left[\begin{array}{cc} U & V\\ Z & {\left(\Theta_{k-1}\right)}^{-1} \end{array}\right]}^{-1}$ U = cI + Ψ(1:n,1:n) V = Ψ(1:n,n+1:end) Z = $V^{T}$ $U^{-1}$ = inv(U) T = $\Theta_{k-1}Z$ Y = U−VT $Y^{-1}$ = inv(Y) % Find the inverse of E = ${\left(\Theta_{k-1}\right)}^{-1}$ − $ZU^{-1}V$ $E^{-1}$ = $\Theta_{k-1}$ + $\left(TY^{-1}\right)\left(V\Theta_{k-1}\right)$ $\Theta_{k}$ = $\left[\begin{array}{cc} Y^{-1} & -U^{-1}VE^{-1}\\ -\Theta_{k-1}ZY^{-1} & E^{-1} \end{array}\right]$ % Use the matrix inversion lemma index = 0 % If maximum eigenvalue is still greater than one, do not enter here else % Algorithm under if condition generally does not work when the number of satellites % changes at *k* % Hence, the following algorithm is used to find Θ then *c* = ($\lambda_{\Psi }^{\max}$ + $\lambda_{\Psi }^{\min}$)/factor % Calculate *c* again for index1 = 1:length(vis_sat_info) % vis_sat_info holds all visible PRN IDs % over *M* epochs % Find epochs over which PRN(index1) is visible. Suppose the number % of epochs is $M_{1}$ $\Theta_{5}$ = inv$\left(cI+\Psi_{\mathrm{sub}}\right)$ % Dimension is $M_{1}\times M_{1}$. See before Equation (30) for the % definition of $\Psi_{\mathrm{sub}}^{i}$ for satellite *i* % Place elements of $\Theta_{5}$ in appropriate entries of Θ corresponding to PRN(index1) end % end of for index1 = 1:length(vis_sat_info) end % end of if(index == 1) factor = factor - 0.2 % Update factor to calculate a new *c* % Calculate $\lambda_{\Omega }^{\max}$ with new Θ end % end of while($\lambda_{\Omega }^{\max}$ > 1)

The preceding algorithm always results in a Θ because there is a value of *c* for which $\lambda_{\Omega }^{\max}$< 1 (see after Equation (40)). The corresponding value of the variable “factor” is $\left(\lambda_{\Psi }^{\max}+\lambda_{\Psi }^{\min}\right)/\left(\lambda_{\Psi }^{\max}-\lambda_{\Psi }^{\min}\right)$. Since $\lambda_{\Psi }^{\max}$ is generally much greater than $\lambda_{\Psi }^{\min}$, the corresponding “factor” is greater than, but close to one. “factor” is started with a value greater than one, and reduced in steps of 0.2 to ensure that *c* is as small as possible. However, the step size cannot be very small because it will increase the number of iterations. It should also be noted that irrespective of the number of visible satellites, the dimension of the matrix to be inverted does not exceed M×M.

## Appendix D. Justification That Γ is Overbounded for $VPE_{2,\;i}^{U}$

First, it is proved that Γ increases as $\lambda_{\tilde{\Theta }}^{\min}$ decreases. Following Equation (56), for a given $T_{th,\;1}$ and $\lambda_{\tilde{\Theta }}^{\min}$, probability $P_{1}$ of the non-central Chi square distribution decreases as $\Gamma^{*}$ increases with Γ. It is proved in [53] that $P_{1}$ increases with $\frac{1}{{\left(\lambda_{\tilde{\Theta }}^{\min}\right)}^{2}}$ for a given $T_{th,\;1}$ and Γ. Therefore, $P_{1}$ vs. Γ curves are decreasing in nature for a given $T_{th,\;1}$ and $\lambda_{\tilde{\Theta }}^{\min}$, and shift upwards as the parameter $\frac{1}{{\left(\lambda_{\tilde{\Theta }}^{\min}\right)}^{2}}$ increases. This implies that Γ increases with $\frac{1}{{\left(\lambda_{\tilde{\Theta }}^{\min}\right)}^{2}}$ (i.e., decreasing $\lambda_{\tilde{\Theta }}^{\min}$) for a given $P_{1}$.

Second, it is justified that the calculated $\lambda_{\tilde{\Theta }}^{\min}$ for $VPE_{2,\;i}^{U}$ is lower than its actual value. From discussions before Equations (37) and (55), it is evident that the actual $\lambda_{\tilde{\Theta }}^{\min}$ is equal to $\lambda_{\Omega }^{\min}$. Next, it is briefly shown that $\lambda_{\Psi \Theta }^{\min}$ > $\lambda_{\Psi ,\;\max\Theta }^{\min}$, where $\Theta \;=\;{\left(cI+\Psi_{\max}\right)}^{-1}$ for $\alpha_{2}$ and $VPE_{2,\;i}^{U}$. The minimum eigenvalue of $\left(\Psi_{\max}\Theta \right)$ is

(A11) $$\lambda_{\Psi ,\;\max\Theta }^{\min}=\frac{1}{\left(c/\lambda_{\Psi ,\;\max}^{\min}+1\right)}$$

if $\Psi_{\max}$ and Ψ are related as $\Psi_{\max}$ = Ψ + ΔΨ, then substituting for Θ and $\Psi_{\max}$, $\lambda_{\Psi \Theta }^{\min}$ can be shown to be

(A12) $$\lambda_{\Psi \Theta }^{\min}=\frac{1}{\left(\lambda_{D}^{\max}+1\right)}$$

where D = $\left(cI+\Delta \Psi \right)\Psi^{-1}$, and the maximum eigenvalue of D is $\lambda_{D}^{\max}$. For $a_{\ell 1}$ of Ψ between $a_{\min}$ and $a_{\max}$, ΔΨ has elements lower than 0.08. Next, $\Psi_{\mathrm{sub}}$ is simulated five thousand times for each (N−M) (number of epochs for $\alpha_{2}$) with uniformly random $a_{\ell 1}$ between $a_{\min}$ and $a_{\max}$, and (N−M) is varied from 1 to 150. With the simulated $\Psi_{\mathrm{sub}}$ Figure A1 justifies that

(A13) $$\lambda_{D}^{\max}\leq \frac{c}{\lambda_{\Psi ,\;\max}^{\min}}$$

Hence,

(A14) $$\lambda_{\Psi \Theta }^{\min}>\frac{1}{\left(c/\lambda_{\Psi ,\;\max}^{\min}+1\right)}=\lambda_{\Psi ,\;\max\Theta }^{\min}$$

By definition, ${{\tilde{S}}^{\prime }}^{T}{\tilde{S}}^{\prime }$ = I and $\left|\right|{\tilde{S}}^{\prime }{\left|\right|}_{2}$ = 1. Next, it was found through simulations that the minimum eigenvalue of ${{\tilde{S}}^{\prime }}^{T}\Psi {\tilde{S}}^{\prime }{{\tilde{S}}^{\prime }}^{T}\Theta {\tilde{S}}^{\prime }$ is greater than that of ${{\tilde{S}}^{\prime }}^{T}\Psi_{\max}{\tilde{S}}^{\prime }{{\tilde{S}}^{\prime }}^{T}\Theta {\tilde{S}}^{\prime }$. The MATLAB code to validate this is provided in the Supplementary Materials (see matlab_prog_min_lambda_theta.m). Thus, the multiplication of ${\tilde{S}}^{\prime }$ preserves the inequality of the preceding equation. The calculated $\lambda_{\tilde{\Theta }}^{\min}$ for $VPE_{2,\;i}^{U}$ is the minimum eigenvalue of ${{\tilde{S}}^{\prime }}^{T}\Psi_{\max}{\tilde{S}}^{\prime }{{\tilde{S}}^{\prime }}^{T}\Theta {\tilde{S}}^{\prime }$ instead of the actual one (i.e., minimum eigenvalue of ${{\tilde{S}}^{\prime }}^{T}\Psi {\tilde{S}}^{\prime }{{\tilde{S}}^{\prime }}^{T}\Theta {\tilde{S}}^{\prime }$ = Ω). Therefore, the calculated $\lambda_{\tilde{\Theta }}^{\min}$ is lower than its actual value, as desired.
